# Adult Circadian Behavior in *Drosophila* Requires Developmental Expression of *cycle*, But Not *period*

**DOI:** 10.1371/journal.pgen.1002167

**Published:** 2011-07-07

**Authors:** Tadahiro Goda, Karolina Mirowska, Jake Currie, Min-Ho Kim, Neethi Varadaraja Rao, Gloribel Bonilla, Herman Wijnen

**Affiliations:** 1 Department of Biology, University of Virginia, Charlottesville, Virginia, United States of America; 2 PhD Program in Biology, University of Virginia, Charlottesville, Virginia, United States of America; University of Texas Southwestern Medical Center, Howard Hughes Medical Institute, United States of America

## Abstract

Circadian clocks have evolved as internal time keeping mechanisms that allow anticipation of daily environmental changes and organization of a daily program of physiological and behavioral rhythms. To better examine the mechanisms underlying circadian clocks in animals and to ask whether clock gene expression and function during development affected subsequent daily time keeping in the adult, we used the genetic tools available in *Drosophila* to conditionally manipulate the function of the CYCLE component of the positive regulator CLOCK/CYCLE (CLK/CYC) or its negative feedback inhibitor PERIOD (PER). Differential manipulation of clock function during development and in adulthood indicated that there is no developmental requirement for either a running clock mechanism or expression of *per*. However, conditional suppression of CLK/CYC activity either via *per* over-expression or *cyc* depletion during metamorphosis resulted in persistent arrhythmic behavior in the adult. Two distinct mechanisms were identified that may contribute to this developmental function of CLK/CYC and both involve the ventral lateral clock neurons (LN_v_s) that are crucial to circadian control of locomotor behavior: (1) selective depletion of *cyc* expression in the LN_v_s resulted in abnormal peptidergic small-LN_v_ dorsal projections, and (2) PER expression rhythms in the adult LN_v_s appeared to be affected by developmental inhibition of CLK/CYC activity. Given the conservation of clock genes and circuits among animals, this study provides a rationale for investigating a possible similar developmental role of the homologous mammalian CLOCK/BMAL1 complex.

## Author Summary

The fruit fly *Drosophila melanogaster* is an excellent model system for studying the internal circadian clocks that animals use for daily time keeping. Since clocks exist and function in animals not only in adults, but also during prior development, the question arises if and how adult circadian rhythms depend on developmental clock circuits and components. To address this question we created transgenic flies in which the essential clock components CLOCK/CYCLE (CLK/CYC) and PERIOD (PER) can be manipulated via environmental temperature. Stopping the clock during development by depleting the negative regulator PER did not prevent restoration of circadian time keeping in the adult. However, a developmental arrest of the clock due to either depletion of the positive regulator CYC or overproduction of PER resulted in a persistent loss of clock-controlled behavior function in adults. Taken together, these observations indicate that adult clock function developmentally requires activity of the CLK/CYC transcription complex rather than a ticking clock. Based on the behavioral, molecular, and anatomical consequences of inhibiting CLK/CYC in circadian pacemaker neurons, we propose that the developmental requirement maps to these cells. It will be interesting to determine whether there is a comparable developmental requirement for the equivalent clock genes in humans.

## Introduction

Circadian clocks are internal daily time keeping mechanisms that allow organisms to anticipate daily environmental rhythms as well as efficiently organize behavioral and physiological functions in a daily schedule. The molecular mechanisms that form the basis for circadian rhythmicity in animals involve interlocked feedback loops controlling gene expression as well as post-translational activities [1], [2]. In both insects and mammals a circadian transcription complex of two basic helix-loop-helix PAS domain transcription factors promotes the rhythmic expression of several of its negative feedback regulators. The fruit fly *Drosophila melanogaster* has emerged as a model system for animal circadian clocks that is both successful and representative. In the clock-bearing cells of *Drosophila* CLOCK/CYCLE (CLK/CYC) acts as the central circadian transcription complex and induces peak expression of a set of transcripts including those for the negative feedback regulators *period* (*per*), *timeless* (*tim*), *vrille* (*vri*), and *clock work orange* (*cwo*) just after dusk [3]–[9]. PER and TIM proteins form a complex with the casein kinase 1ε ortholog DOUBLETIME (DBT), in which TIM helps protect PER from destabilization by DBT-mediated phosphorylation [10]–[12]. PER-containing complexes enter the nucleus around midnight and trigger repression of CLK/CYC activity [5], [13]–[16], VRI acts as a transcriptional repressor for the *Clk* gene [9], [17], and CWO reduces CLK/CYC activity by competitively binding CLK/CYC-regulated promoter elements [4], [7], [8].

The circadian clock circuits are linked to synchronizing input pathways as well as output pathways that signal time-of-day information to downstream biological functions. The extensive interconnectedness of the molecular circadian cycle complicates identification of the order of its events. We reasoned that the development of transgenic flies with conditional circadian clock function, in which the circadian cycle could be arrested or started at will, would help distinguish direct from indirect effects and determine sequential steps in circadian pathways. Moreover, transgenic flies with conditionally titratable transcription of a clock component would allow molecular, cellular, and behavioral circadian phenotypes to be determined over a range of expression levels. Finally, flies with conditionally controlled clock function would allow separation of developmental and adult functions of clock genes. Based on these arguments we created conditionally rhythmic transgenic *Drosophila* strains.

In the present study, we describe the generation of transgenic flies in which clock function becomes conditional on account of temperature-dependent rescue of the *per^01^* or *cyc^01^* mutations or temperature-dependent mis-expression of *per*. Moreover, we made use of these flies to experimentally determine the developmental requirements for a functional circadian clock as well as the individual clock components PER and CYC. We confirmed and extended previously published observations [18], [19] indicating that developmental rescue of arrhythmia in *per^01^* mutants is not needed for restoration of circadian rhythms in adults. However, developmental mis-expression of *per* or failure to developmentally rescue the *cyc^01^* mutation led to persistent adult arrhythmia. In particular, CLK/CYC function during the pupal and pharate adult stages was associated with adult clock function. Our results suggest two distinct mechanisms underlying the developmental requirement for CLK/CYC function: (1) *cyc* expression contributes cell-type-autonomously in the ventral lateral neurons (LN_v_s) to the formation of peptidergic dorsal projections containing the neuropeptide PIGMENT DISPERSING FACTOR (PDF), which are thought to be important for adult circadian behavior and (2) CLK/CYC activity during development enables normal clock gene expression rhythms in the adult LN_v_s.

## Results

### Transgenic flies with conditional rescue of *per^01^* in clock-bearing cells

**Figure 1:**
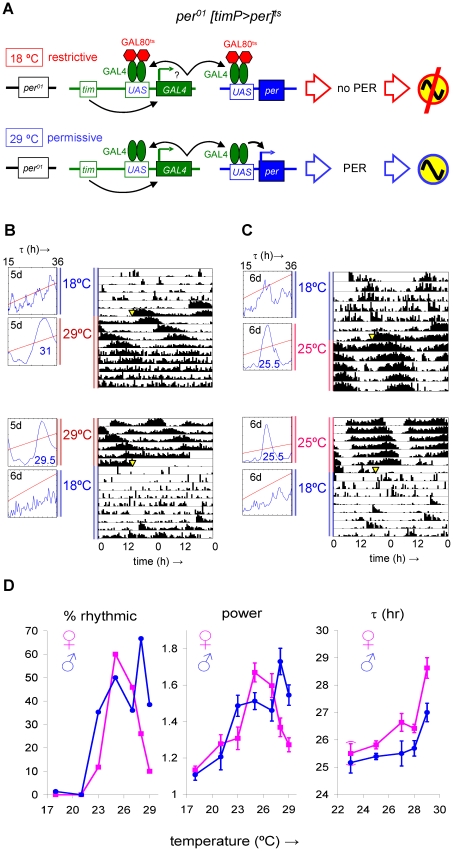
Conditional transgenic rescue of *per^01^* arrhythmic behavior. Introduction of *tim(UAS)-Gal4*, *UAS-per*, and *tubP-Gal80^ts^* transgenes in a *per^01^* genetic background resulted in conditional circadian clock function, with virtually no rescue of circadian locomotor behavior at the restrictive temperature (18°C) and approximation of wild-type circadian behavior at permissive temperatures (23–29°C). (A) The lack of rescue at 18°C is explained by inhibition of GAL4-mediated expression of transgenic *per* mediated by the temperature sensitive GAL4 repressor GAL80^ts^. At permissive temperatures the modulating effect of GAL80^ts^ is reduced allowing GAL4 expressed in clock-bearing cells to induce *per* and rescue circadian clock function. (B,C) Flies with a *y per^01^ w; tim(UAS)-Gal4/tubPGal80^ts^; UAS-per/+* genotype, abbreviated as *per^01^ [timP>per]^ts^*, raised at ambient temperature (∼23°C) were monitored for adult locomotor activity sequentially at restrictive and permissive conditions (see Materials and Methods). The large diagrams in (B) and (C) are double-plotted actograms representing the median locomotor activity for 8 female flies. Yellow triangles indicate the time of the temperature shifts. The small diagrams are chi-square periodograms based on the first 5 or 6 full days at the first (top) or second (bottom) experimental condition. Circadian period lengths detected at the permissive conditions are indicated in large blue type-face. Note the absence of strong circadian rhythms at 18°C. The considerably lengthened period and progressive weakening of rhythms observed at 29°C are likely attributable to excessive *per* expression. (D) Temperature-dependent conditional rescue of the percentage of rhythmic flies, relative rhythmic power, and period length in *per^01^ [timP>per]^ts^* flies. Chi-square periodogram analysis of circadian locomotor behavior in DD was performed for 5-day intervals at the indicated temperatures. The three panels show changes as a function of environmental temperature in the percentage of rhythmic flies, relative rhythmic power (across both rhythmic and weakly rhythmic flies), and circadian period length for rhythmic flies. Error bars represent the Standard Error of the Mean (SEM).

We made use of the temporal and regional gene expression targeting (TARGET) system [20] to create transgenic flies in which the essential clock components CYC and PER were expressed conditionally in relevant spatiotemporal patterns. The TARGET system combines the binary GAL4/UAS system [21] that allows transgenic expression to be directed spatiotemporally by a promoter of interest via the intermediate regulator GAL4 with a ubiquitously expressed GAL80^ts^ gene, which encodes a temperature sensitive inhibitor of GAL4. As a result, the TARGET system permits GAL4-mediated transgenic expression at high temperatures (e.g., 29°C), but progressively restricts it at lower temperatures. First, we generated transgenic flies that conditionally rescued the arrhythmic *per^01^* phenotype [22] by introducing a GAL4-driver transgene directing expression in all clock-bearing cells (*tim(UAS)-Gal4*) [9] along with a GAL4-responsive per cDNA expression construct (*UAS-per*) [23] and a transgene ubiquitously expressing GAL80^ts^ (*tubP-Gal80^ts^*) [20] in a *per^01^* genetic background [22] (see Figure 1A). The resulting genotype is abbreviated, here, as *per^01^[timP>per]^ts^*. As expected, clock-controlled phenotypes such as behavioral rhythmicity, relative rhythmic power and period length were readily and significantly modulated by environmental temperature in these flies (Figure 1B–1D, Figures S1 and S2). Robust circadian rhythms in locomotor activity were virtually absent at a restrictive temperature (18°C), but rescued to varying degrees over a range of higher temperatures (21–29°C) (Figure 1B–1D, Figures S1 and S2). The circadian period length observed at 29°C was significantly longer than those at 25°C, 27°C, and 28°C for females and those at 23°C, 25°C and 28°C for males (Figure 1D, Figure S2A, S2C; Welch test and post-hoc Games-Howell analysis). It is noteworthy that the decrease in rhythmicity and relative rhythmic power and the increase in circadian period length found at the highest experimental temperature of transgenic induction (29°C) were also observed as a result of transgenic *per* over-expression in a wild-type background (see below). In comparison with wild-type controls *per^01^[timP>per]^ts^* flies were much less rhythmic at 18°C (or 29°C), but at 25°C both genotypes showed comparable percentages of rhythmic, weakly rhythmic, and arrhythmic flies (Figure S3). At permissive temperatures the most consistent difference in the behavior of *per^01^[timP>per]^ts^* flies relative to wild-type controls was a significantly longer circadian period length increased by 2 h or more (Figure S3B–S3D).

Next, we examined clock-controlled molecular responses in *per^01^[timP>per]^ts^* flies released from restrictive (17 or 18°C) to permissive conditions (25°C). As expected, transgenic *per* expression was strongly induced in adult fly heads following this transition (Figure S4A, S4B). In addition, the *Clk*, *tim*, *vri*, *cwo*, *Par-domain Protein 1 (Pdp1)*, and *Slow-poke binding protein (Slob)* clock-controlled transcripts showed relative expression responses that appeared consistent with their circadian phase relationships in wild-type heads. Nevertheless, the amplitude of the observed expression responses in clock-controlled genes was reduced relative to previously reported amplitudes of circadian oscillation in wild-type heads [4], [7]–[9], [24]–[27]. Thus, upon transfer to permissive conditions, rescue of molecular circadian oscillations in adult *per^01^[timP>per]^ts^* heads, unlike behavioral rhythms, appeared to be incomplete. This discrepancy might be explained by a selective restoration of high-amplitude clock gene expression rhythms in clock neurons. We, therefore, examined circadian transcript responses in dissected adult brains of *per^01^[timP>per]^ts^* flies released under permissive conditions. However, molecular amplitudes in adult brains were comparable to those previously seen in adult heads (cf Figure S4A and S4C) suggesting incomplete restoration of molecular circadian rhythms in both peripheral clocks and the neural clock circuit. Since different time points in the Northern and Quantitative Reverse Transcriptase PCR (qRT-PCR) experiments of Figure S4 come from different samples of individual flies incomplete synchrony in the experimental population may have also contributed to the detection of relatively shallow transcript rhythms.

### Adult circadian locomotor behavior does not require a functioning circadian clock or rescue of *per^01^* during prior development

**Figure 2:**
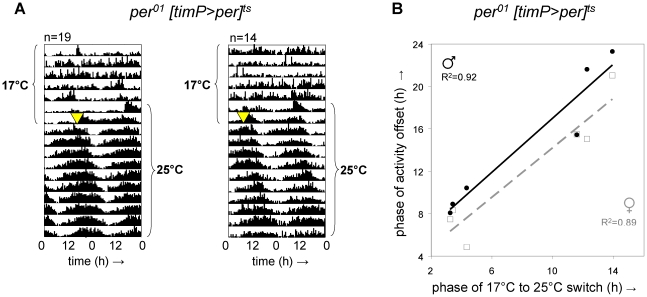
Adult circadian behavior does not require developmental rescue of *per^01^*. Circadian locomotor behavior was readily restored in *per^01^ [timP>per]^ts^* flies raised under restrictive conditions (17°C LL) upon transfer of adults from restrictive (17°C DD) to permissive conditions (25°C DD). Moreover, the phase of circadian behavior was determined by the phase of transfer. (A) Double-plotted actograms representing average locomotor activity at 17°C DD and subsequent 25°C DD. The left panel represents data for 19 female flies transferred to the permissive condition at reference phase hour 12.5, whereas the right panel corresponds to the activity of 14 female flies switched at reference phase 3.25. Note the phase relationship between subsequent behavioral rhythms and the time of transfer (marked by a yellow triangle). (B) The phase of the offset of circadian locomotor activity at 25°C is plotted separately for median data from 6 groups of male and 6 groups of female flies as a function of the phase of the 17°C to 25°C switch along with trend lines and associated correlation coefficients (2-tailed test significance: p<0.005 for females, p<0.003 for males).

To test if developmental expression of *per* in clock-bearing cells was required for adult clock function, we raised *per^01^[timP>per]^ts^* flies at 17°C in constant light (LL) until adulthood and examined behavioral rhythms at restrictive (17°C) and subsequent permissive (25°C) conditions in constant darkness (DD). Consistent with the hypothesis that rescue of circadian clock function in *per^01^* flies can be achieved when *per* expression is restricted to clock-bearing cells in the adult, we observed restoration of circadian locomotor rhythms immediately following transition to permissive conditions (Figure 2A, Figure S5). Although adult *per^01^[timP>per]^ts^* flies failed to show strongly rhythmic locomotor behavior at the restrictive temperature in DD, a subset of individual flies did exhibit residual weak rhythms under these conditions (Figure S2). Nevertheless, we do not believe that the observed behavioral rescue in adults depends on residual clock function during the prior exposure to the restrictive temperature for two reasons: (1) The phase of the restored rhythms is determined by the phase of the prior switch from restrictive to permissive conditions rather than the phase of the light/dark transition associated with the start of the behavioral experiment (Figure 2) and (2) our experiments included developmental exposure to LL, which is associated with both behavioral and molecular arrhythmia as well as severely reduced PER expression levels [13], [28]. Therefore, we conclude that there is no developmental requirement for either a functioning clock mechanism or expression of *per* in the clock-bearing cells in order to allow circadian clock function in adult flies.

### Over-expression of *per* during metamorphosis disrupts adult circadian behavior

**Figure 3:**
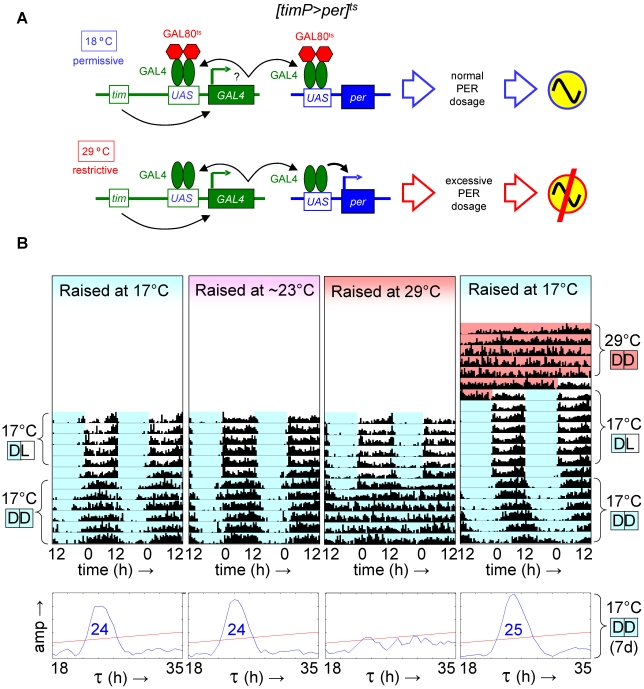
Developmental over-expression of *per* disrupts adult circadian behavior, while the phenotype of adult *per* over-expression is reversible. Transgenic flies with conditional over-expression of *per* exhibited a reversible loss of circadian behavior when temporarily shifted to restrictive conditions (29°C) as adults, but showed long-term behavioral arrhythmia when similarly exposed to restrictive conditions during development. (A) Flies with a *y tubPGal80^ts^ w/(FM7c or Y); tim(UAS)-Gal4; UAS-per* genotype, abbreviated as *[timP>per]^ts^*, show rhythmic locomotor behavior at the permissive temperature when *per* over-expression is prevented by GAL80^ts^, but not at the restrictive temperature when GAL80^ts^ is ineffective and excessive levels of PER prevent circadian clock function. (B) The top four panels are double-plotted actograms representing median locomotor activity data for groups of female *[timP>per]^ts^* flies during LD and subsequent DD conditions at the permissive temperature (17°C DD). The two actograms on the left illustrate that developmental exposure to 17°C or ∼23°C has no obvious effect on adult circadian behavior, while the data in the third panel from the left reflect the loss of adult locomotor activity rhythms following development at the restrictive temperature (29°C). The right-most actogram illustrates the reversible behavioral phenotype of 17°C-raised flies that where shifted to the restrictive temperature as adults (29°C DD data included at the top) prior to the rest of the experiment. The white, light blue, and red background colors in the actograms represent 17°C light, 17°C dark, and 29°C dark conditions, respectively. The lower four panels are chi-square periodograms for the median data reflecting circadian rhythmicity in 17°C DD. Single significant (p<0.01) period lengths in the circadian range (18–35 h) are indicated in large blue type-face.

**Figure 4:**
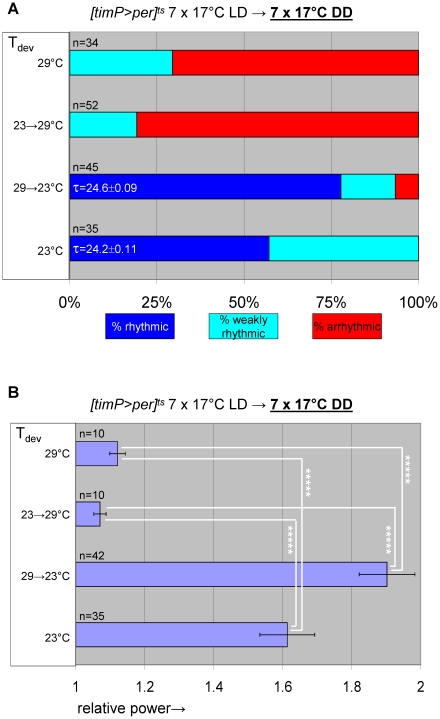
Over-expression of *per* in clock-bearing cells during metamorphosis disrupts locomotor activity rhythms in adults. Circadian behavior of adult *[timP>per]^ts^* flies under permissive conditions (17°C DD) was strongly rhythmic for flies raised at ambient temperature (∼23°C) or flies transferred from 29°C to ambient temperature as wandering larvae or prepupae. However, flies exposed to restrictive (29°C DD) conditions throughout development or during the pupal and pharate adult stages exhibited mostly arrhythmic adult locomotor behavior. (A) Stacked bar diagram representing the percentages of female flies with rhythmic, weakly rhythmic, or arrhythmic adult behavior at permissive conditions (17°C DD). Prior to measurement of adult locomotor activity at 17°C LD and subsequent 17°C DD (analyzed here) flies were raised at the indicated temperatures (T_dev_): 23°C, 23→29°C (transferred as wandering larvae or prepupae from 23°C to 29°C), 29→23°C (transferred as wandering larvae or prepupae 29°C to 23°C), and 29°C. The numbers (n) of flies included for each condition are indicated as well as the average (±SEM) circadian period length for rhythmic flies. Chi-square analysis indicated a highly significant (p<10^−23^) association between developmental temperature and the percentages of rhythmic, weakly rhythmic, and arrhythmic adults. (B) Bar diagram of the average (±SEM) relative rhythmic power observed among the rhythmic plus weakly rhythmic flies for each developmental condition. The number of flies included in this analysis (n) is indicated for each condition. The Welch test statistic indicated a highly significant association (p<10^−13^) of relative rhythmic power with developmental condition. Significant differences found by post-hoc Games-Howell tests for pairwise comparisons of developmental treatments indicated by (*****) represent p values smaller than 10^−5^.

**Figure 5:**
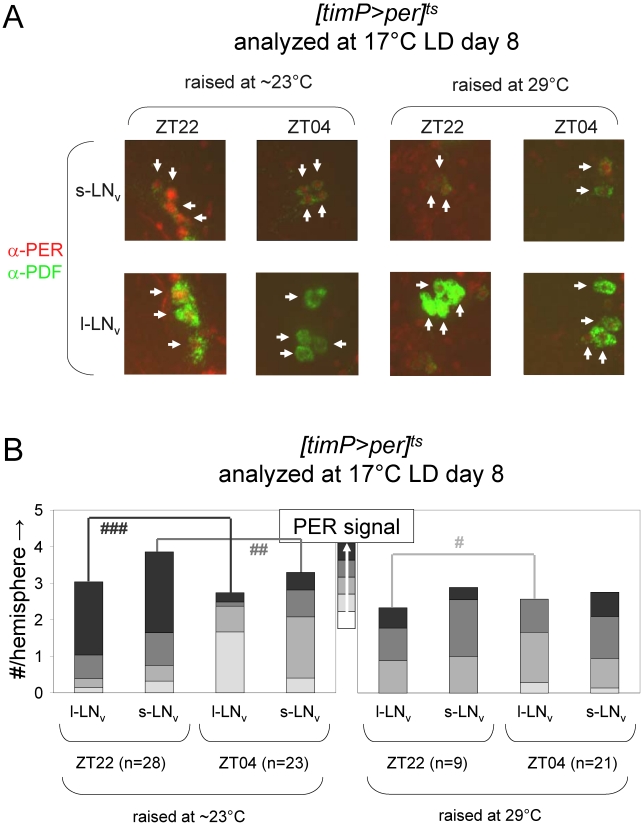
Developmental over-expression of *per* affects molecular rhythms in adult clock neurons. Comparative immunofluorescence analyses indicate that developmental *per* over-expression affects circadian PER protein rhythms in the PDF-expressing adult LN_v_s. (A) *[timP>per]^ts^* flies raised at either restrictive (29°C) or permissive (∼23°C) conditions were transferred as adults to 17°C LD conditions and harvested 2-h before lights-on (ZT22) on day 8 and 4-h after lights-on (ZT4) on day 9. Brains were dissected and subjected to immunofluorescence staining using antibodies directed against the PER and PDF proteins. Representative images illustrate the effects of both daily phase and developmental treatment on PER expression for PDF-expressing LN_v_ cells (arrows). (B) Semi-quantitative analysis of the immunofluorescence signal for PER in adult PDF neurons. The cumulative height of the bars indicate the average number per brain hemisphere of l-LNv and s-LNv detected by PDF staining, whereas the segments with increasingly darker shades of gray represent the relative prevalence of cells with no, very faint, faint, moderate, or strong levels of PER signal, respectively. The numbers of brain hemispheres is indicated (n). Significant associations of PER signal level and daily phase found by Chi-square analyses indicated by (#), (##), and (###) represent p values smaller than 10^−6^, 10^−10^, and 10^−17^, respectively.

Given the ability to restore adult clock function from a circadian cycle arrest due to PER depletion, we were wondering whether circadian cycle arrests associated with excess PER expression were equally reversible. This question was addressed experimentally with the help of transgenic flies, in which *per* was conditionally over-expressed to high levels in clock-bearing cells due to the introduction of the *tim(UAS)-Gal4*, *UAS-per*, and *tubP-Gal80^ts^* transgenes in the presence of a wild-type *per* gene. Flies homozygous for the autosomal *tim(UAS)-Gal4* and *UAS-per* insertions with a single X-chromosomal *tubP-Gal80^ts^* transgene (abbreviated as *[timP>per]^ts^*) showed conditional clock function with robust rhythms, relative rhythmic power, and only marginally increased period lengths at permissive (17°C) conditions and behavioral arrhythmia (females) or dramatically reduced rhythms (males) at the restrictive (29°C) conditions (Figure 3, Figure 4, Figures S6 and S7). Loss of behavioral rhythms during prolonged exposure of adults to the restrictive condition could be reversed by returning the flies to the permissive (17°C) condition (Figure 3B, Figure S7). However, comparable exposure to restrictive conditions during development resulted in irreversible adult arrhythmia (Figure 3B, Figure 4, Figures S6 and S7) for both genders. To identify the developmental phase of sensitivity to PER over-expression flies were transferred from a permissive ambient temperature (∼23°C) to 29°C or vice versa at different points during development and then analyzed for behavioral rhythmicity as adults. When exposure to restrictive conditions occurred prior to the pupal stage it did not obviously affect the percentages of flies exhibiting rhythmic, weakly rhythmic or arrhythmic adult behavior or the relative power of the detected rhythms (Figure 4, Figure S6). However, when flies were exposed to the restrictive temperature throughout the pupal and pharate adult stages, adult locomotor rhythms were clearly inhibited (Figure 4, Figure S6). Therefore, it appears that *per* over-expression in pupal/pharate adult clock cells irreversibly affects adult circadian behavior. One possible explanation for the observed effect of developmental *per* mis-expression on adult behavior might be the persistence of abnormally high levels of PER protein into adulthood. However, PER is known to be an unstable protein and even a 7-d exposure to 12-h light/12-h dark/ (LD) cycles at the permissive (17°C) temperature did not allow subsequent restoration of behavioral rhythms in DD. Moreover, immunofluorescence analyses of clock neurons exposed to developmental PER over-expression did not reveal a continued increase in adult PER expression. Instead, the persistent behavioral arrhythmia of *[timP>per]^ts^* flies raised at 29°C and exposed to 17°C LD for 7 d as adults appeared to be matched by blunted circadian rhythms of PER expression in the PDF-expressing ventral lateral neurons (Figure 5). No gross morphological defects in clock neurons (including LN_v_, LN_d_, and DN cell bodies and LN_v_ projections) were apparent in these experiments (see Figure S8). Thus, our results indicate that excess PER activity in clock cells during metamorphosis negatively affects both adult circadian locomotor activity and molecular rhythms in adult clock neurons.

### Depletion of *cyc* expression during metamorphosis disrupts adult circadian behavior

**Figure 6:**
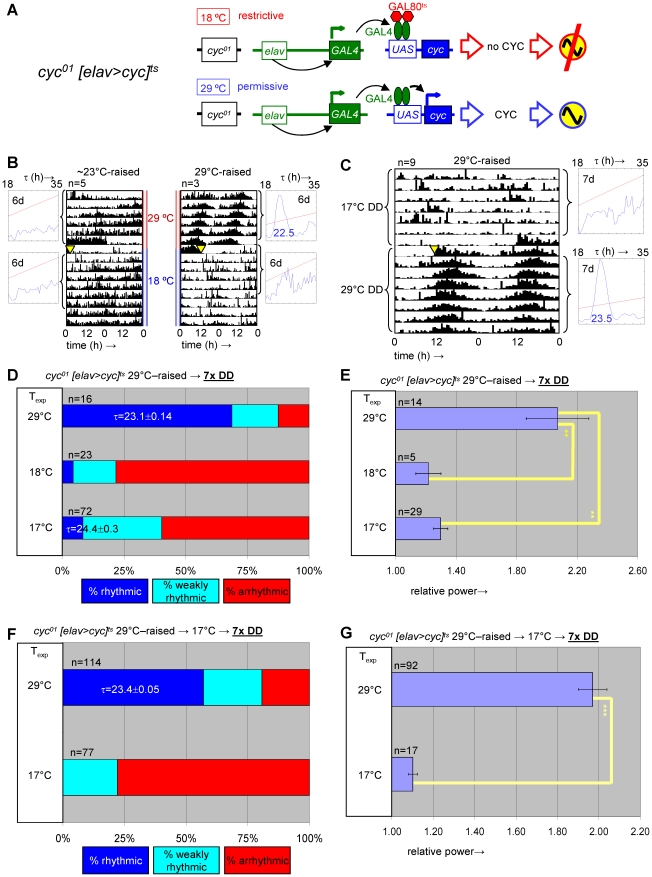
Developmental depletion of *cyc* disrupts adult circadian behavior, while the phenotype of adult depletion of *cyc* is reversible. (A) *elav^C155^::Gal4; UAS-cyc/CyO; cyc^01^ tubPGal80^ts^* flies, here abbreviated as *cyc^01^ [elav>cyc]^ts^*, conditionally rescue *cyc^01^* in postmitotic neurons at 29°C, but not lower temperatures (17–25°C). (B) Example actograms illustrate average locomotor activity at 29°C DD and subsequent 18°C DD conditions for females raised at restrictive (∼23°C) or permissive temperature (29°C), respectively. (C) The actogram shows behavioral arrhythmicity at 17°C for 29°C-raised females and subsequent rescue of circadian locomotor activity at 29°C. (B–C) Associated chi-square periodograms show strong behavioral rhythms only observed for 29°C-raised flies at 29°C. (D–G) Quantitative analysis of adult circadian behavior in 29°C-raised *cyc^01^ [elav>cyc]^ts^* flies at either permissive (29°C) versus two restrictive conditions (17°C, 18°C) (D,E) or permissive (29°C) versus restrictive (17°C) conditions following adult exposure to restrictive conditions (≥3 days 17°C) (F,G). The stacked bar diagrams (D,F) represent the percentages of 29°C-raised females with rhythmic, weakly rhythmic, or arrhythmic locomotor behavior. Rhythmicity was determined for individual flies by chi-square periodogram analysis of 7 d intervals at the indicated temperatures in constant darkness. The average (±SEM) circadian period length is indicated for rhythmic flies. Chi-square analyses indicated highly significant associations between experimental temperature and the percentages of rhythmic, weakly rhythmic, and arrhythmic adults [p<10^−7^ (D); p<10^−17^ (F)]. The bar diagrams (E,G) correspond to the average (±SEM) relative rhythmic power observed among the rhythmic plus weakly rhythmic flies for each experimental condition. The number of flies included in this analysis (n) is indicated for each condition. (E) The Welch test statistic indicated a significant association (p<10^−2^) of relative rhythmic power with experimental condition. Significant differences found by post-hoc Games-Howell tests for pairwise comparisons of developmental treatments indicated by (**) represent p values smaller than 10^−2^. (G) A significant association (p<10^−3^) of relative rhythmic power with experimental condition was found by the Mann-Whitney rank-sum test.

**Figure 7:**
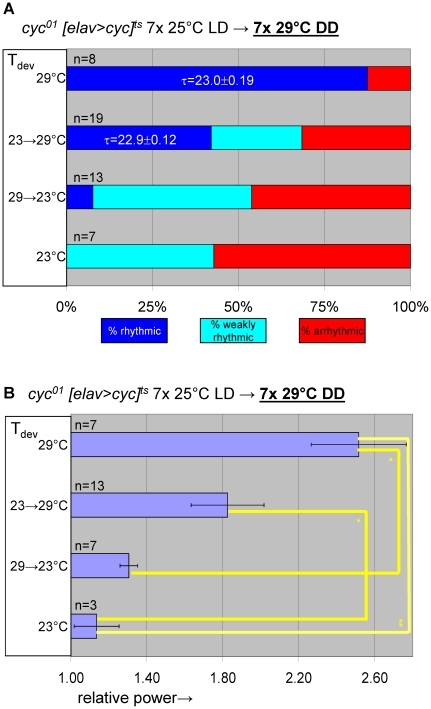
Transgenic rescue of *cyc^01^* behavioral arrhythmia in adults depends on developmental *cyc* expression during metamorphosis. Adult circadian behavior of 29°C-raised *cyc^01^ [elav>cyc]^ts^* flies was strongly rhythmic under permissive conditions (29°C DD). Substantial rescue of adult locomotor rhythms was also observed for *cyc^01^ [elav>cyc]^ts^* flies raised at ambient temperature (∼23°C) and transferred to 29°C as wandering larvae or prepupae. However, flies exposed to restrictive (∼23°C) conditions throughout development or during the pupal and pharate adult stages exhibited weakly rhythmic or arrhythmic adult locomotor behavior. (A) Stacked bar diagram representing the percentages of female flies with rhythmic, weakly rhythmic, or arrhythmic adult behavior at permissive conditions (29°C DD) following prior exposure to 7 LD days at 25°C. Prior to measurement of adult locomotor activity during 25°C LD and subsequent 29°C DD (analyzed here) flies were raised at the indicated temperatures (T_dev_): 23°C, 23→29°C (transferred as wandering larvae or prepupae from 23°C to 29°C), 29→23°C (transferred as wandering larvae or prepupae 29°C to 23°C), and 29°C. The numbers (n) of flies included for each condition are indicated as well as the average (±SEM) circadian period length for rhythmic flies. Chi-square analysis indicated a significant (p<10^−2^) association between developmental temperature and the percentages of rhythmic, weakly rhythmic, and arrhythmic adults. (B) Bar diagram of the average (±SEM) relative rhythmic power observed among the rhythmic plus weakly rhythmic flies for each developmental condition. The number of flies included in this analysis (n) is indicated for each condition. The Welch test statistic indicated a significant association (p<10^−2^) of relative rhythmic power with developmental condition. Significant differences found by post-hoc Games-Howell tests for pairwise comparisons of developmental treatments indicated by (*) and (**) represent p values smaller than 0.05 and 10^−2^, respectively.

Based on PER's known function as a negative regulator of CLK/CYC circadian transcription complexes the adult phenotypes associated with developmental PER over-expression are likely attributable to inhibition of CLK/CYC activity. We tested this hypothesis by determining whether adult circadian locomotor behavior required prior developmental expression of the essential clock component CYC. To this aim we generated transgenic flies that conditionally expressed *cyc* in postmitotic neurons by combining the *elav^C155^::Gal4* driver element [29] with *UAS-cyc* [30], and *tubP-Gal80^ts^* transgenes in a *cyc^01^* background. The resulting flies, here referred to as *cyc^01^ [elav>cyc]^ts^*, showed conditional rescue of rhythmic adult locomotor activity when raised at the permissive temperature for *cyc^01^* rescue (29°C) (Figure 6, Figure S9). Ambient temperature (∼23°C), which acted as a mostly permissive condition for *per^01^ [timP>per]^ts^* flies (see Figure 1D, Figure S2, above) represented a restrictive condition for the *cyc^01^ [elav>cyc]^ts^* strain. This discrepancy is likely attributable to differences either in the amount of GAL4 protein produced in the relevant clock neurons in each of these strains or the level of GAL4-directed transgenic expression that is required to achieve behavioral rescue. Exposure of *cyc^01^ [elav>cyc]^ts^* flies to the restrictive temperature during metamorphosis, severely affected adult behavioral rhythms at the permissive temperature (Figure 6B, Figure 7, Figure S10). Therefore, depletion of *cyc* expression during metamorphosis, indeed, phenocopies the adult behavioral defects of *per* mis-expression during metamorphosis.

### Selective inhibition of *cyc^01^* rescue in the PDF-expressing clock neurons disrupts adult locomotor rhythms as well as cell-type-specific neuro-anatomy and molecular rhythms

**Figure 8:**
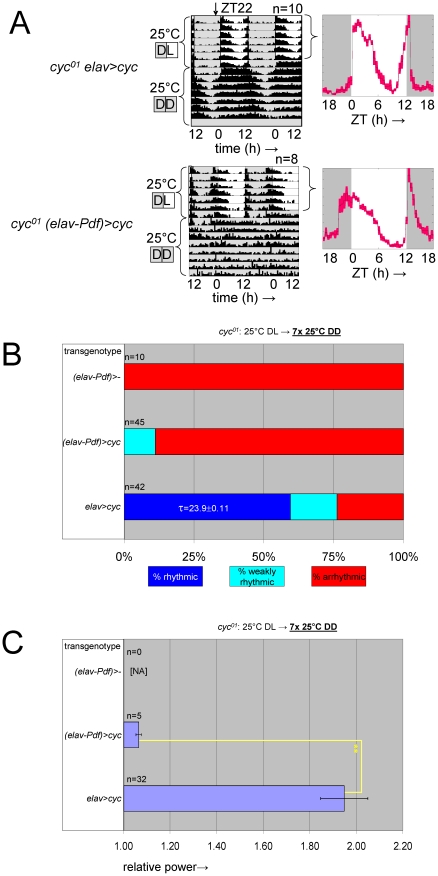
Selective inhibition of *cyc^01^* rescue in the PDF-expressing clock neurons results in behavioral circadian phenotypes. (A) Example actograms (left) and LD activity profiles (right) representing median locomotor behavior for female *elav^C155^::Gal4; UAS-cyc/CyO; cyc^01^* flies (abbreviated as *cyc^01^ elav>cyc*), which are rescued for *cyc* expression in postmitotic neurons, versus female *elav^C155^::Gal4; UAS-cyc/Pdf-Gal80; cyc^01^* flies (abbreviated as *cyc^01^ (elav-Pdf)>cyc*), in which transgenic *cyc* rescue is selectively blocked in the PDF-expressing clock neurons. The activity profiles represent average daily activity (±SEM) indicated by the black line and red shading for the median locomotor activity of *cyc^01^ elav>cyc* and *cyc^01^ (elav-Pdf)>cyc* flies in the presence of LD cycles. Note that *cyc^01^ (elav-Pdf)>cyc* flies exhibit loss of free running rhythms in DD as well as increased activity in anticipation of lights-on and loss of activity in anticipation of lights-off in LD. (B) Stacked bar diagram representing the percentages of female flies with rhythmic, weakly rhythmic, or arrhythmic adult behavior at 25°C DD. Along with *cyc^01^ elav>cyc* and *cyc^01^ (elav-Pdf)>cyc* flies, the experiment also included non-rescued control flies with an *elav^C155^::Gal4; CyO/Pdf-Gal80; cyc^01^* genotype (abbreviated as *cyc^01^ (elav-Pdf)>-*). For each transgenic combination with *cyc^01^* the number of flies (n) as well as the average (±SEM) circadian period length for rhythmic flies are indicated. Chi-square analysis indicated a highly significant (p<10^−9^) association between genotype and the percentages of rhythmic, weakly rhythmic, and arrhythmic adults. (C) Bar diagram of the average (±SEM) relative rhythmic power observed among the rhythmic plus weakly rhythmic female flies for each genotype. The number of flies included in this analysis (n) is indicated for each condition. Because all *cyc^01^ (elav-Pdf)>-* flies were arrhythmic, a Mann-Whitney rank-sum test was performed to compare the effect on relative rhythmic power of the other two genotypes. As indicated, relative rhythmic power was significantly reduced in *cyc^01^ (elav-Pdf)>cyc* flies compared to *cyc^01^ elav>cyc* flies (**; p<10^−2^).

To further explore the role of CLK/CYC expression in the PDF-positive LN_v_s in ensuring normal adult circadian behavior and neuro-anatomy we created flies in which rescue of *cyc^01^* in postmitotic neurons (by *elav^C155^::Gal4* and *UAS-cyc*) was selectively blocked in the PDF-expressing LNvs with the help of a *Pdf-Gal80* transgene [31] that expresses the GAL4 inhibitor GAL80 specifically in these cells. The behavioral phenotype of the resulting transgenic flies, indicated as *cyc^01^ (elav-Pdf)>cyc*, consists of an altered daily locomotor activity profile in the presence of light/dark cycles that includes extended activity in anticipation of lights-on, but reduced activity in anticipation of lights-off (Figure 8A, Figure S11A) and a loss of sustained rhythmicity in constant darkness (Figure 8A–8C; Figure S11). In contrast, control *cyc^01^* rescue flies lacking the *Pdf-Gal80* element (*cyc^01^ elav>cyc*) showed strong behavioral rhythms in constant darkness as well as evening activity in anticipation of the lights-off transition (Figure 8A–8C; Figure S11). The *cyc^01^ (elav-Pdf)>cyc* phenotype is clearly different from that of flies with ablated PDF-expressing LN_v_s or defective expression of the PDF neuropeptide [32], which also lack consolidated rhythms in constant darkness but show the opposite effect on anticipation of the lights-on and lights-off transitions. The persistence of morning anticipation, which is thought to be attributable to PDF signaling from the s-LN_v_s [33] suggests that residual PDF expression and function persisted in s-LN_v_s with a *cyc^01^* circadian cycle arrest. Moreover, it is insightful to compare the behavior of *cyc^01^ (elav-Pdf)>cyc* flies to previously published observations for flies, in which rescue of the *per^01^* mutation in clock-bearing cells was blocked in the PDF-expressing clock neurons (*per^01^ (elav-Pdf)>per*) [31]. The behavior reported for *per^01^ (elav-Pdf)>per* flies resembles that of our *cyc^01^ (elav-Pdf)>cyc* flies with respect to the persistence of morning anticipation as well as reduced rhythmicity in constant darkness [31]. However, loss of evening anticipation appears to be unique to the *cyc^01^*-based as opposed to the *per^01^*-based arrest of the LN_v_s. Thus, *cyc*-depleted LN_v_s seemed to delay the generation of an evening activity signal by the neural clock circuits. It is possible that a slow rhythmic component in the disorganized clock circuit of *cyc^01^ (elav-Pdf)>cyc* flies is responsible for this delay in evening activity. Consistent with this notion, the few (weakly) rhythmic *cyc^01^ (elav-Pdf)>cyc* flies represented in Figure 8 and Figure S11 exhibited residual long period rhythms (τ for females: 25.1±1.24 h, n = 5; τ for males 24.4±0.83 h, n = 17).

**Figure 9:**
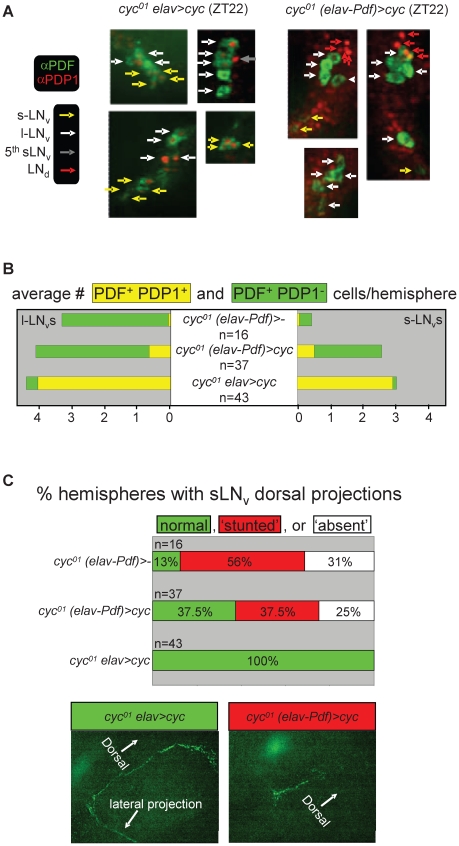
Selective inhibition of *cyc^01^* rescue in the PDF-expressing clock neurons results in cell-type–specific neuro-anatomical and molecular circadian phenotypes. Comparative immunofluorescence analysis of PDF and PDP1 expression in the clock neurons of *cyc^01^ elav>cyc* and *cyc^01^ (elav-Pdf)>cyc* flies. (A) Example micrographs illustrating selective inhibition of PDP1 expression in the PDF-expressing LNvs of *cyc^01^ (elav-Pdf)>cyc* but not *cyc^01^ elav>cyc* flies at ZT22. Whole mount adult brains for both genotypes were stained with antibodies directed against PDF (green) and PDP1 (red). s-LN_v_, l-LN_v_, LN_d_, and 5^th^ s-LN_v_ clock neurons are indicated by yellow, white, red, and gray arrows, respectively. (B) Semi-quantitative analysis of the number of PDP1-positive and PDP1-negative PDF-expressing LN_v_s at ZT22 per brain hemisphere for *cyc^01^ (elav-Pdf)>-, cyc^01^ (elav-Pdf)>cyc*, and *cyc^01^ elav>cyc* flies. The numbers (n) of brain hemispheres analyzed are indicated for each genotype. Chi-square analyses found highly significant associations between genotype and PDP1 expression in both s-LN_v_s (p<10^−31^) and l-LN_v_s (p<10^−55^). (C) Semi-quantitative analysis of neuro-anatomical defects in the dorsal PDF-expressing LN_v_ projections. Stacked bar diagram (top panel) representing the percentages of *cyc^01^ (elav-Pdf)>-*, *cyc^01^ (elav-Pdf)>cyc*, and *cyc^01^ elav>cyc* brain hemispheres with normal, stunted, or absent PDF-positive dorsal projections. The numbers (n) of brain hemispheres analyzed are indicated for each genotype. Chi-square analysis indicated a highly significant association between genotype and dorsal projection phenotype (p<10^−5^). The lower two panels represent examples of normal and stunted PDF-expressing dorsal projections, from *cyc^01^ elav>cyc* and *cyc^01^ (elav-Pdf)>cyc* brains, respectively.

Next, we compared molecular and neuro-anatomical phenotypes of the PDF-expressing LN_v_s in *cyc^01^ (elav-Pdf)>cyc* flies with those of controls lacking either the *Pdf-Gal80* (*cyc^01^ elav>cyc*) or the *UAS-cyc* transgenes (*cyc^01^ (elav-Pdf)>-*). Flies of these three genotypes were raised at ambient temperature and entrained as adults to LD cycles at 25°C. Brains were harvested 2 h prior to lights-on (ZT22) and stained using antibodies against PDF and the PDP1. The *Pdp1* gene is a direct target gene for CLK/CYC and mutations in *Clk* or *cyc* strongly affect PDP1 protein expression in larvae and adults [26]. Indeed, *cyc^01^ (elav-Pdf)>-* flies, which completely lack CLK/CYC function, exhibited greatly reduced PDP1 expression in their LN_v_s at ZT22. Consistent with previous studies [34], s-LN_v_s in *cyc^01^ (elav-Pdf)>-* brains also showed a reduced PDF signal in the s-LN_v_s and mostly abnormal or missing PDF-positive dorsal projections (Figure 9). These phenotypes were to a large extent rescued in *cyc^01^ elav>cyc* flies, which not only exhibited PDP1 expression in virtually all PDF-expressing LN_v_s (and other clock neurons) at ZT22, but also presented with normal PDF-expression levels and dorsal LN_v_ projections (Figure 9). The additional introduction of *Pdf-Gal80* in the *cyc^01^ (elav-Pdf)>cyc* genotype, resulted in cell-type-specific phenotypes that included down-regulation of PDP1 in virtually all LN_v_s with detectable PDF expression as well as a reduction in the number of s-LNvs with detectable PDF expression (see Figure 9A, 9B). Moreover, PDF-positive sLN_v_ dorsal projections were either abnormal or missing from most *cyc^01^ (elav-Pdf)>cyc* brains (see Figure 9C), although there is a formal possibility that PDF-negative sLN_v_ projections, which would not have been detectable in these experiments, still extended to the dorsal protocerebrum. For other clock neurons no obvious differences were detected in numbers and PDP1 expression levels between the *cyc^01^ (elav-Pdf)>cyc* brains and the rescued *cyc^01^ elav>cyc* controls.

## Discussion

We created transgenic flies with conditional clock function, in which expression of the essential clock components CYC and PER was induced or repressed in relevant spatiotemporal patterns. In *per^01^ [timP>per]^ts^* flies, which conditionally rescue the *per^01^* mutation, clock function was conditional and readily reversible. Moreover, adult circadian behavior was restored in flies raised under restrictive conditions. In earlier studies conducted by Ewer and colleagues widespread transgenic expression of *per* under control of a *heat-shock protein 70* (*hsp70*) promoter was shown to partially rescue the *per^01^* mutation resulting in restoration of behavioral rhythms at an abnormally long period length. These long period rhythms could be generated in a conditional manner even when induction was restricted to the adult phase [18], [19]. In the present study we targeted expression of transgenic *per* specifically to clock-bearing cells and achieved a more complete conditional rescue of the *per^01^* phenotype that did not require developmental *per* expression.

Although circadian behavior of *per^01^ [timP>per]^ts^* flies at 25°C showed rhythmicity comparable to that observed for wild-type flies, period lengths were at least 2 h longer than those of wild-type flies and molecular rhythms showed a relatively low amplitude. One key difference between the molecular clock circuits in *per^01^ [timP>per]^ts^* at the permissive temperature and those of wild-type flies is the constitutively high level of *per* mRNA expression in the transgenically rescued flies, which could contribute to the increased circadian period length and blunted molecular rhythms in *per^01^ [timP>per]^ts^* flies. Wild-type flies exhibit a trough in *per* transcript levels in the early morning that may facilitate subsequent down-regulation of PER protein levels and optimal induction of CLK/CYC-regulated genes [35]. The lack of a trough in *per* mRNA expression in the conditionally rescued flies could account for a delay in the turnover of PER protein in the morning and, therefore, a lengthened period and blunted CLK/CYC activity. This hypothesis also explains apparent discrepancies with previous reports, in which increased *per* gene dosage was associated with a shortened circadian period length [36] and decreased *per* dosage or expression resulted in longer circadian period lengths [22], [37], [38]. As long as *per* expression shows strong circadian regulation the timing of PER nuclear entry and PER-mediated transcriptional repression is predicted to be advanced by the introduction of one or two additional copies of the wild-type *per* gene and delayed by a reduction in *per* dosage, while neither manipulation is predicted to strongly affect subsequent PER turnover.

Adult circadian behavior was also conditional and reversible in *[timP>per]^ts^* flies, which exhibit temperature-dependent over-expression of *per*. However, developmental over-expression of *per* during metamorphosis was associated with irreversible behavioral arrhythmia in adults. Likewise, depletion of *cyc* expression during the metamorphosis in *cyc^01^ [elav>cyc]^ts^* flies resulted in disruption of adult circadian locomotor behavior under permissive conditions. Both increased levels of PER and decreased levels of CYC negatively regulate CLK/CYC activity. The CLK/CYC heterodimer functions as the central transcriptional regulator in the *Drosophila* clock and its activity critically depends on the presence of both CLK and CYC [3], [5], [6]. Loss of functional *cyc* expression in the *cyc^01^* mutant results in both molecular and circadian arrhythmia and constitutively low expression levels for CLK/CYC-regulated target genes [6], whereas PER acts as a negative regulator of CLK/CYC activity by binding and inactivating the CLK/CYC complex [5], [15], [39]. The arrhythmic locomotor behavior and molecular arrhythmia in the clock neurons observed as a result of *per* over-expression [23], [40] are, therefore, interpreted to result from constitutive inhibition of CLK/CYC.

Adult behavioral arrhythmia in *[timP>per]^ts^* or *cyc^01^ [elav>cyc]^ts^* flies raised under permissive conditions was reversible (see Figure 3B, Figure 6C, 6F, 6G, Figures S7, S9C, S9D, above). However, exposures to restrictive conditions of comparable duration resulted in long-term after-effects only when they occurred during development and, particularly, during the pupal and pharate adult stages. We, therefore, attribute the effects of circadian arrests during development in *[timP>per]^ts^* or *cyc^01^ [elav>cyc]^ts^* flies on adult circadian behavior to a developmental requirement for CLK/CYC function beyond its immediate role in maintaining daily time keeping. The requirement for CLK/CYC activity, but not clock function per se may indicate that one or more transcriptional CLK/CYC targets play a role in enabling adult circadian locomotor behavior. Such targets would likely be expressed constitutively along with other CLK/CYC-regulated genes in conditionally arrested *per^01^ [timP>per]^ts^* flies, but constitutively down-regulated in circadian arrests due to low CLK/CYC activity.

A central question that remains is what mechanism links developmental CLK/CYC activity to adult circadian behavior. Our experiments indicate that both clock neuron anatomy and the molecular oscillator itself may be involved. Previously published studies of constitutively arrhythmic alleles of the *Clk* and *cyc* genes have documented a reduction in PDF expression as well as neuro-anatomical defects in the LN_v_s [34] that could be associated with a developmental role for the CLK/CYC transcription factor. By selectively blocking transgenic rescue of *cyc^01^* in the PDF-expressing clock neurons we show, here, that the reduction of PDF expression and PDF-positive dorsal projections from the s-LN_v_s is a cell-type specific phenotype. PDF is known to play an important role in mediating clock-controlled behavior in both LD and DD conditions. The PDF-producing s-LN_v_s project towards the dorsal protocerebrum as do DN1, DN2, DN3, and LN_d_ clock neurons, suggesting that the dorsal s-LN_v_ projections may play an important part in signaling across the neural clock circuits [41]. In this context, it may be relevant that expression of the PDF RECEPTOR in ‘E’ cells, a subset of clock neurons including DN1s and LN_d_s [31], has been associated with circadian control of locomotor activity [42]. Moreover, the axonal terminals of the dorsal s-LNv projections undergo clock-controlled rhythms in remodeling that may play a role in circadian signaling [43]. Nevertheless, the observed developmental requirement for CLK/CYC activity also appears to involve mechanisms other than PDF-mediated signaling for the following reasons. First, developmental over-expression of PER resulted in persistent adult arrhythmia, but did not lead to a loss of PDF-positive dorsal projections from the s-LN_v_s (see Figure S8). Second, while developmental suppression of CLK/CYC activity uniformly affected the behavior of adult flies (Figure 4, Figure 7, Figures S6, S7, S10) constitutive depletion of CYC from the PDF-expressing neurons resulted in a variable phenotype in the s-LN_v_ dorsal projections (see Figure 9C). Third, the light/dark activity pattern of *cyc^01^ (elav-Pdf)>cyc* flies (Figure 8A, Figure S11A) was strikingly different from that of *Pdf^01^* flies or flies from which the PDF-expressing cells have been ablated [32], suggesting that PDF signaling persisted in *cyc*-depleted LN_v_s in spite of the defects in PDF-positive dorsal projections.

In principle, neuro-anatomical defects affecting intercellular connectivity rather than cell-autonomous clock function could lead to behavioral phenotypes due to asynchrony among the clock neurons or the loss of output signals. Indeed, apparent separation of molecular and behavioral phenotypes has been reported previously for genetic manipulation of CLK/CYC function [44], [45]. It may be particularly relevant that rescue of the *per^01^* phenotype in the PDF-expressing clock neurons restores rhythmic behavior [46], while rescue of *cyc^01^* in the same cells restores molecular, but not behavioral rhythms [45]. However, our experimental results also provide support for developmental phenotypes at the level of the adult molecular clock circuits. Our immunofluorescence expression analyses indicated that the molecular clock circuits in the adult PDF-expressing clock neurons were affected by developmental over-expression of PER. PDF-expressing LN_v_s in adults that were behaviorally arrhythmic due to developmental PER over-expression exhibited adult PER expression with an altered daily profile, but not necessarily at excessively high levels. Future studies may determine the degree to which neuro-anatomical and molecular phenotypes are linked and help determine the effect of intercellular connectivity in the neural clock circuit on the function of molecular circadian rhythms in individual clock neurons.

## Materials and Methods

### *Drosophila* stocks

Flies were raised on standard yeast cornmeal agar food either at ambient temperature (observed to range between 22°C and 24°C) or other experimental temperatures as specified. The conditional *per^01^* rescue flies indicated as *per^01^ [timP>per]^ts^* in Figure 1, Figure 2 and Figures S1, S2, S3, S5 consisted of male and female *y per^01^ w; tim(UAS)-Gal4/tubPGal80^ts^; UAS-per/+* offspring from a cross between stable lines *y per^01^ w; tim(UAS)-Gal4* and *y per^01^ w; tubPGal80^ts^; UAS-per*. These stocks were created by combining the previously described *per^01^* [22], *tim(UAS)-Gal4* [9], *tubPGal80^ts^* [20], and *UAS-per* [23] genetic elements. Flies with conditional over-expression of *per* in clock-bearing cells (*[timP>per]^ts^* in Figure 3, Figure 4, Figure 5, and Figures S6, S7, S8) were obtained from a genetically stable *y tubPGal80^ts^ w/FM7c; tim(UAS)-Gal4; UAS-per* stock as females heterozygous for *FM7c* and non-*FM7c* males. The insertion site of the *tubPGal80^ts^* transgene in this stock appears to be associated with homozygous female lethality. An X-chromosomal period-lengthening allele present in the genetic background of the original *tubPGal80^ts^* stocks was avoided during the creation of the *[timP>per]^ts^* stock by recombination with a control *y w* chromosome. Flies with conditional rescue of *cyc^01^* (*cyc^01^ [elav>cyc]^ts^* in Figure 6, Figure 7 and Figures S9, S10) were obtained from a stable *elav^C155^::Gal4; UAS-cyc/CyO; cyc^01^ tubPGal80^ts^* stock as males and females heterozygous for *CyO.* The *elav^C155^::Gal4* [29], *UAS-cyc* [30], *cyc^01^* [6], and *tubPGal80^ts^* [20] elements used to create this stock had al been described previously. The *cyc^01^* rescue line *elav^C155^::Gal4; UAS-cyc/CyO; cyc^01^* (*cyc^01^ elav>cyc* in Figure 8, Figure 9 and Figure S11) was created as a stable stock, whereas the selective *cyc^01^* rescue genotype *elav^C155^::Gal4; UAS-cyc/Pdf-Gal80; cyc^01^* and the unrescued control genotype *elav^C155^::Gal4; CyO/Pdf-Gal80; cyc^01^* (respectively, *cyc^01^ (elav-Pdf)>cyc* and *cyc^01^ (elav-Pdf)>-* in Figure 8, Figure 9 and ) were obtained in offspring from a cross of *elav^C155^::Gal4; UAS-cyc/CyO; cyc^01^* flies with *elav^C155^::Gal4; Pdf-Gal80; cyc^01^* flies. The *Pdf-Gal80* element used in the latter two genotypes has also been characterized previously [31].

### Locomotor behavior assays

Using previously described protocols [47], locomotor activity was monitored for individual adult flies of both genders in glass tubes on standard sugar agar media including 0.07% Tegosept (Genesee Scientific) using the Drosophila Activity Monitoring System (TriKinetics). Experiments were conducted in incubators kept at 70% relative humidity in 12 h L∶ 12 h D or DD conditions using white fluorescent light with an approximate intensity of 450 µW/cm^2^ during the L condition. Due to lack of space only analyses for female flies are shown in Figure 1BC, Figure 4, Figure 6D–6G, Figure 7, and Figure 8; the corresponding analyses for male flies are found in Figures S1, S6, S9, S10, and S11, respectively.

### Statistical analyses of locomotor activity data

Individual, experimental average, and experimental median activity records, as well as periodic activity profiles, and chi-square periodograms were generated using ClockLab Software (ActiMetrics). Actograms (Figure 1BC, Figure 2A, Figure 3B, Figure 6B and 6C, Figure 8A, Figures S1, S3A–S3C, S11A) were double-plotted with a resolution of half-hour intervals. Each row represents a 2-day interval of Zeitgeber Time (ZT, with ZT0 as the time of lights-on; during LD) or Circadian Time (CT; during DD), of which the second day is repeated as the first day on the next row. Chi-square periodograms (Figure 1BC, Figure 3B, Figure 6B and 6C, Figures S1 and S3A–S3C) were used to represent the experimental signal (amplitude) observed for a range of period lengths (τ, x-axis) relative to threshold values associated with a p<0.01 significance (red line). Analyses of the percentages of rhythmic, weakly rhythmic, and arrhythmic flies (Figure 1D, Figure 4A, Figure 6D and 6F, Figure 7A, Figure 8B, Figures S2AC, S3D, S5AC, S6A, S7A, S7B, S7D, S7E, S9A, S9C, S10A, S11B) were based on chi-square periodogram statistics for locomotor activity rhythms of individual flies. For period lengths in the circadian range (∼15–36 h) detected with a significance of p<0.01 relative rhythmic power was calculated by dividing the detected peak amplitude by the significance threshold value at the same period length. Flies were classified based on their values of relative rhythmic power as rhythmic (>1.5) or weakly rhythmic ([1,1.5]) and flies without significant periodicity in the circadian range were considered arrhythmic. Chi-square analyses for association of genotype or experimental protocol with the relative distribution of rhythmic, weakly rhythmic, or arrhythmic behavior were conducted using Microsoft Excel (Microsoft). Next, statistical analyses were performed using SPSS software (IBM) to detect associations between the relative rhythmic power values of rhythmic and weakly rhythmic flies with experimental conditions (Figure 4B, Figure 6E and 6G, Figure 7B, Figures S2BD, S5BD, S6B, S7CF, S9BD, S10B) or genotypes (Figure 8C, Figure S11C). In virtually all cases Levene's test indicated that homogeneous variances could not be assumed. Therefore, the Welch test statistic with Games-Howell post-hoc analysis was used to test for significant differences in relative rhythmic power among different genotypes and treatments. When only two conditions were compared the non-parametric Mann-Whitney rank-sum test was performed. Average (Figure 2A, Figure 6B and 6C, Figure S3A, S3B, S3C) and median (Figure 1B and 1C, Figure 3B, Figure 8A, Figures S1, S11A) activity records that emphasize reproducible features of rhythmic locomotor activity measured in individual flies were created without prior normalization from the raw individual activity records on a point-by-point basis. For representation in illustrative double-plotted actograms we generally used median activity records, which are less susceptible to skewing by outliers and show discrete numbers of events per half-hour bin, but when a better resolution of data with relatively low activity counts was preferred average activity records were used instead. Average daily or circadian activity profiles representing records of median or average activity ± the Standard Error of the Mean (SEM) were generated across included days under entraining (Figure 8A, Figure S11A) or free running conditions (S3, for phase determination in Figure 2B), respectively. The phase of the offset of circadian activity (Figure 2B) was determined from the activity profiles by interpolation as described previously [47]. Error bars throughout the manuscript represent SEM, except in cases where less than three observations were made. The parentheses surrounding individual error bars in Figure 1B (right-hand panel), Figure S7F, and Figure S9B indicate that these represent the range of two observations, instead.

### Northern analysis

Extraction of total RNA from approximately 100 µl adult heads per time point using the guanidinium thiocyanate/cesium chloride method and subsequent Northern analysis were conducted according to previously published protocols [48], [49]. Quantitative analysis of the radioactive signals on the blots was conducted with a Storm 840 Phosphorimager (GE healthcare) and the resulting data was graphed using Microsoft Excel (Microsoft Corporation). Five independent time course experiments were conducted addressing the transcript responses observed in the adult head upon transfer of *per^01^ [timP>per]^ts^* flies from restrictive to permissive conditions. A representative example is shown in Figure S4A.

### Quantitative reverse transcriptase PCR (qRT-PCR) analysis

Flies for the conditions of interest were harvested onto ice, and either adult heads or brains were dissected on a chilled platform and transferred to guanidinium thiocyanate buffer. DNAse I-digested total RNA was obtained from the heads or brains using the RNAqueous4PCR kit (Ambion). Aliquots of the RNA samples were then analyzed with the SuperScript III Platinum SYBR Green One-Step qPCR Kit (Invitrogen) using experimental primer pairs designed to specifically amplify fragments of the circadian *per*, *tim*, *vri*, and *cwo* transcripts, the transgenic *UAS-per* transcript or the *rp49* control transcript. Expression levels measured on a SmartCycler system (Cepheid) relative to *rp49* were determined using the comparative Cycle threshold (Ct) method [50].

### Immunofluorescence analysis

Adult brains were dissected, fixed, and stained for immunofluorescence analysis according to standard protocols [51]. Imaging was conducted with a spinning disk confocal microscope. Brains from *[timP>per]^ts^* flies raised under restrictive versus permissive conditions were probed with primary antibodies against PDF (mouse monoclonal; DSHB) as well as PER (rabbit polyclonal; [52]), whereas brains from *cyc^01^ elav>cyc* and *cyc^01^ (elav-Pdf)>cyc* flies were stained with antibodies to PDF as well as PDP1 (rabbit polyclonal;[26]) and then visualized with fluorescently labeled secondary antibodies (Alexa-488 for PDF, Alexa-568 for PER or PDP1).

## Supporting Information

Figure S1 Conditional transgenic rescue of *per^01^* arrhythmic behavior in male flies. (A,B) Median actogram and periodogram analyses for representative groups of male *per^01^ [timP>per]^ts^* flies (n = 8 in each case) that were obtained in parallel with the data for female flies in Figure 1B and 1C. Locomotor rhythms in DD are strongly reduced at the restrictive temperature (18°C) compared to permissive conditions (25°C, 29°C) and the lengthening period and weakening of rhythms in the long term observed at 29°C are likely attributable to the effects of excessive *per* expression.
(PDF)

Figure S2 Quantitative analysis of adult circadian behavior in *per^01^ [timP>per]^ts^* flies across a range of temperatures. Both male and female adult *per^01^ [timP>per]^ts^* flies exhibited temperature-dependent locomotor activity rhythms. The stacked bar diagrams (A,C) represent the percentages of female (A) and male (C) *per^01^ [timP>per]^ts^* with rhythmic, weakly rhythmic, or arrhythmic adult locomotor behavior at different experimental temperatures. Rhythmicity was determined for individual flies by chi-square periodogram analysis of 5-d intervals at the indicated temperatures in constant darkness. The numbers (n) of flies included for each condition are indicated as well as the average (±SEM) circadian period length for rhythmic flies. Chi-square analyses indicated highly significant associations (females p<10^−15^; males p<10^−18^) between experimental temperature and the percentages of rhythmic, weakly rhythmic, and arrhythmic adults. The bar diagrams (B,D) correspond to the average (±SEM) relative rhythmic power observed among the rhythmic plus weakly rhythmic female (B) and male (D) flies for each experimental temperature. The number of flies included in this analysis (n) is indicated for each condition. Welch test analyses indicated highly significant associations (females p<10^−9^; males p<10^−10^) of relative rhythmic power with experimental condition. Significant differences found by post-hoc Games-Howell tests for pairwise comparisons of developmental treatments indicated by (*), (**), (***), (****), and (*****) represent p values smaller than 0.05,10^−2^,10^−3^,10^−4^, and 10^−5^, respectively.
(PDF)

Figure S3 Comparison of temperature-dependent locomotor behavior of flies with conditional rescue of *per^01^* to that of wild-type flies. (A–C) Analyses of locomotor behavior of *per^01^ [timP>per]^ts^* (conditional *per^01^* rescue) and Canton-S (wild-type) flies in 5-d intervals in constant darkness at 18°C (A), 25°C (B), and 29°C (C). Top row: Actograms for representative individual flies showing locomotor activity across Circadian Time (CT) in constant darkness. Middle row: Chi-square periodogram data for the 5-d intervals and flies represented in the top row. Significant circadian period lengths (τ; p<0.01) are indicated (blue type face) as well as classification into rhythmic (R), weakly rhythmic (WR) and arrhythmic (AR) flies. Bottom row: Average circadian activity profiles were generated from the average data of 8 (or 7) flies over the 5-d intervals. The profiles are plotted relative to the circadian period lengths detected in the average data. If no significant periodicity was found, (A: column 1,2) the profiles were plotted over 24 h. (D) The stacked bar diagram represents a comparison of the distribution of rhythmic, weakly rhythmic, or arrhythmic *per^01^ [timP>per]^ts^* flies at 18°C, 25°C, and 29°C relative to wild-type (Canton-S) controls. The numbers (n) of flies included for each condition are indicated as well as the average (±SEM) circadian period length for rhythmic flies. Chi square analyses revealed significant differences in rhythmicity at 18°C and 29°C, but not 25°C, with p-values smaller than 10^−4^ and 10^−5^ indicated by (****) and (*****), respectively. In addition, Mann-Whitney rank-sum tests comparing circadian period length at 25°C and 29°C between rhythmic *per^01^ [timP>per]^ts^* and wild-type control flies indicated significantly longer periods (p<0.001) for the former.
(PDF)

Figure S4 Conditional induction of clock gene mRNA expression in adult *per^01^ [timP>per]^ts^* heads and brains. (A) Total RNA extracted from adult fly heads collected immediately prior to or at 6,12,18,24,30, or 36 h after a shift from restrictive 17°C to permissive 25°C conditions in DD was analyzed on Northern blots for the expression of the indicated clock-controlled transcripts. The data for each transcript represent expression ratios relative to an internal control (*rp49*) that were normalized to the experimental average. The signal for *per* encompassed both the endogenous *per^01^* and transgenic *per* transcripts and exhibited a temperature-dependent induction. Clock-controlled transcript profiles exhibited shallow amplitudes but maintained the expected relative phase relationships, with peak expression of *tim*, *vri*, *cwo*, and *Slob* peaking ahead of *Pdp1*, which in turn was phase-advanced relative to *Clk*. Similar observations were made for several independent experiments. (B) Quantitative Reverse Transcriptase PCR (qRT-PCR) analysis was performed using primers that were (right panel) or were not (left panel) selective for transgenic *per* relative to native *per^01^* transcripts. Total RNA was extracted from the heads of *per^01^ [timP>per]^ts^* flies as well as controls lacking the *tim(UAS)-Gal4* driver transgene (*per^01^ [->per]^ts^*) immediately before and at 15 and 30 h after a 18°C DD (restrictive) to 25°C DD (permissive) shift. Signals were quantified using the cycle threshold method [50] relative to *rp49* transcript. Total *per^01^/per* transcript and transgenic *per* transcript were induced at 15 h in *per^01^ [timP>per]^ts^* heads approximately five-fold and more than forty-fold, respectively, while *per* transcript levels in *per^01^ [->per]^ts^* flies remained constitutively low. (C) Total RNA was extracted from dissected adult brains of *per^01^ [timP>per]^ts^* flies immediately prior to as well as at 15 and 30 h after a 18°C DD to 25°C DD shift. Signals in adult brains for clock genes (*cwo*, *tim*, *vri*) and total *per^01^/per* (relative to rp49 and normalized to experimental average) showed only modest responses after the shift to permissive conditions.
(PDF)

Figure S5 Rhythmicity and power of circadian locomotor activity are conditionally rescued in *per^01^ [timP>per]^ts^* adults raised at restrictive conditions. Adult locomotor behavior was examined in female (A,B) and male (C,D) adult *per^01^ [timP>per]^ts^* flies raised under restrictive conditions (17°C LL) at restrictive (17°C DD) and subsequent permissive conditions (25°C DD). (A,C) Stacked bar diagrams representing the percentages of flies with rhythmic, weakly rhythmic, or arrhythmic adult behavior during 7 d at 17°C DD versus 7 d at 25°C DD. The numbers (n) of flies included for each condition are indicated as well as the average (±SEM) circadian period length for rhythmic flies. Chi-square analyses indicated a highly significant association between experimental condition and the percentages of rhythmic, weakly rhythmic, and arrhythmic females (p<10^−6^) and males (p<10^−4^). (B,D) Bar diagrams of the average (±SEM) relative rhythmic power observed among the rhythmic plus weakly rhythmic flies for each developmental condition. The number of flies included in this analysis (n) is indicated for each condition. Statistical analyses (Mann-Whitney rank-sum test) indicated a significant association between relative rhythmic power and experimental condition in both females (**; p<10^−2^) and males (***; p<10^−3^).
(PDF)

Figure S6 Over-expression of *per* in clock-bearing cells during metamorphosis disrupts locomotor activity rhythms in adult males. Circadian behavior of adult *[timP>per]^ts^* males under permissive conditions (17°C DD) was determined by the temperature of pupal and pharate adult development. While males raised at ambient temperature (∼23°C) or transferred from 29°C to ambient temperature as wandering larvae or prepupae were strongly rhythmic as adults at the permissive temperature, males exposed to restrictive (29°C DD) conditions throughout development or during the pupal and pharate adult stages exhibited arrhythmic adult locomotor behavior. (A) Stacked bar diagram representing the percentages of males with rhythmic, weakly rhythmic, or arrhythmic adult behavior at permissive conditions (17°C DD). Prior to measurement of adult locomotor activity during 17°C LD and subsequent 17°C DD (analyzed here) flies were raised at the indicated temperatures (T_dev_): 23°C, 23→29°C (transferred as wandering larvae or prepupae from 23°C to 29°C), 29→23°C (transferred as wandering larvae or prepupae 29°C to 23°C), and 29°C. The numbers (n) of flies included for each condition are indicated as well as the average (±SEM) circadian period length for rhythmic flies. Chi-square analysis indicated a highly significant (p<10^−13^) association between developmental temperature and the percentages of rhythmic, weakly rhythmic, and arrhythmic adults. (B) Bar diagram of the average (±SEM) relative rhythmic power observed among the rhythmic plus weakly rhythmic male flies for each developmental condition. The number of flies included in this analysis (n) is indicated for each condition. The Welch test statistic indicated a highly significant association (p<10^−8^) of relative rhythmic power with developmental condition. Significant differences found by post-hoc Games-Howell tests for pairwise comparisons of developmental treatments indicated by (****) and (*****) represent p values smaller than 10^−4^ and 10^−5^.
(PDF)

Figure S7 Developmental over-expression of *per* affects rhythmicity and power of adult circadian behavior. Circadian locomotor activity was analyzed for *[timP>per]^ts^* adult females (A–C) and males (D–F) that were raised either under restrictive conditions (29°C) or permissive conditions (∼23°C) or transferred from restrictive to permissive conditions, or vice versa, at the wandering larva/prepupa stage. (A,B,D,E) Stacked bar diagrams representing the percentages of rhythmic, weakly rhythmic, or arrhythmic flies at restrictive conditions (29°C DD) (A,D) or subsequent permissive conditions (17°C DD after 7 d at 17°C LD) (B,E). Prior to analysis of adult locomotor activity flies were raised at the indicated temperatures (T_dev_): 23°C, 23→29°C (transferred as wandering larvae/prepupae from 23°C to 29°C), 29→23°C (transferred as wandering larvae/prepupae 29°C to 23°C), and 29°C. Average(±SEM) circadian period length is indicated for rhythmic flies. (A) Females were essentially arrhythmic at 29°C regardless of developmental treatment (Chi-square p = 0.25). (D) In contrast, males, which have a relatively higher dosage of *Gal80^ts^* (see Materials and Methods), showed a highly significant association between developmental temperature and rhythmicity at 29°C (Chi-square p<10^−5^) and exhibited long-period rhythms at 29°C following development under permissive conditions. (B,E) Both genders demonstrated a highly significant correlation between developmental treatment and rhythmicity at 17°C during the last step of the experiment (Chi-square females p<10^−9^, males p<10^−5^) when flies exposed to permissive conditions during metamorphosis showed circadian period lengths approaching 24-h. (C,F) Bar diagrams of the average(±SEM) relative rhythmic power observed among the rhythmic plus weakly rhythmic flies at 17°C DD for each developmental condition. For 29°C-raised males (n = 2) the range rather than the SEM is indicated in (F). The Welch test statistic indicated significant associations between relative rhythmic power at 17°C and developmental treatment (p<10^−4^ in both female and males). Significant differences found by post-hoc Games-Howell tests for pairwise comparisons of developmental treatments indicated by (**), (***) and (****) represent p values smaller than 10^−2^, 10^−3^ and 10^−4^, respectively.
(PDF)

Figure S8 DN and LNd subsets of clock neurons as well as PDF-positive dorsal projections persist after developmental *per* over-expression. DN and LNd clock neurons were detected by anti-PER immunofluorescence in adult brains of both ∼23°C and 29°C-raised *[timP>per]^ts^* flies (experiment described in Figure 5). Representative images are shown. PER signal is weaker in the brain of the ∼23°C-raised fly because it was taken from the ZT4 time point, while the image for the 29°C-raised fly is from ZT22. LN_v_ clock neurons are identified based on co-staining with anti-PDF antibody. Note the presence of PDF-stained dorsal projections from the LN_v_s in both brains (indicated by the white arrows).
(PDF)

Figure S9 Clock function is conditional in adult *cyc^01^ [elav>cyc]^ts^* males raised at the permissive condition. Quantitative analysis of adult circadian behavior in 29°C-raised *cyc^01^ [elav>cyc]^ts^* male flies at either permissive (29°C) versus two restrictive conditions (17°C, 18°C) (A,B) or permissive (29°C) versus restrictive (17°C) conditions following adult exposure to restrictive conditions (≥3 days 17C) (C,D). The stacked bar diagrams (A,C) represent the percentages of 29°C-raised male *cyc^01^ [elav>cyc]^ts^* flies with rhythmic, weakly rhythmic, or arrhythmic adult locomotor behavior. Rhythmicity was determined for individual flies by chi-square periodogram analysis of 7 d intervals at the indicated temperatures in constant darkness. The numbers (n) of flies included for each condition are indicated as well as the average (±SEM) circadian period length for rhythmic flies. Chi-square analyses indicated significant associations between experimental temperature and the percentages of rhythmic, weakly rhythmic, and arrhythmic adults (p<10^−3^ (A); p<10^−13^ (C)). The bar diagrams (B,D) correspond to the average (±SEM) relative rhythmic power observed among the rhythmic plus weakly rhythmic flies for each experimental condition. The number of flies included in this analysis (n) is indicated for each condition. Because there were only two observations for rhythmic/weakly rhythmic males at 18°C the range rather than the average±SEM is indicated. (B) Welch test analyses indicated a significant association of relative rhythmic power with experimental condition (p<10^−2^). A significant difference was found by post-hoc Games-Howell test for pairwise comparison of males at 29°C versus 18°C (**; p<0.02). (D) Statistical analysis (Mann-Whitney rank-sum) indicated a significant association between relative rhythmic power and experimental condition (***; p<10^−3^).
(PDF)

Figure S10 Association between developmental *cyc* expression and transgenic rescue of behavioral arrhythmia in adult males. (A) Stacked bar diagram representing the percentages of male flies with rhythmic, weakly rhythmic, or arrhythmic adult behavior at permissive conditions (29°C DD) following prior exposure to 7 LD days at 25°C. Flies were raised at the indicated temperatures (T_dev_): 23°C, 23→29°C (transferred as wandering larvae or prepupae from 23°C to 29°C), 29→23°C (transferred as wandering larvae or prepupae 29°C to 23°C), and 29°C prior to analysis of adult locomotor activity during 25°C LD and subsequent 29°C DD conditions. The numbers (n) of flies included for each condition are indicated as well as the average (±SEM) circadian period length for rhythmic flies. Although the trends in rhythmicity relative to developmental conditions resemble the associations found in females chi-square analysis did not demonstrate a significant (p = 0.11) association in males. (B) Bar diagram of the average (±SEM) relative rhythmic power observed among the rhythmic plus weakly rhythmic flies for each developmental condition. The number of flies included in this analysis (n) is indicated for each condition. Again, trends of relative rhythmic power relative to developmental condition resemble the associations found in female flies, but the Welch test statistic did not demonstrate a significant association of relative rhythmic power with developmental condition (p = 0.15).
(PDF)

Figure S11 Male *cyc^01^ (elav-Pdf)>cyc* flies show behavioral arrhythmia. (A) Example actograms (left) and LD activity profiles (right) representing median locomotor behavior for male *cyc^01^ elav>cyc* versus *cyc^01^ (elav-Pdf)>cyc* flies. Note that *cyc^01^ (elav-Pdf)>cyc* flies exhibit loss of free running rhythms in DD as well as increased activity in anticipation of lights-on and loss of activity in anticipation of lights-off in LD. (B) Stacked bar diagram representing the percentages of *cyc^01^ elav>cyc*, *cyc^01^ (elav-Pdf)>cyc*, and *cyc^01^ (elav-Pdf)>-* male flies with rhythmic, weakly rhythmic, or arrhythmic adult behavior at 25°C DD. The number of flies (n) as well as the average (±SEM) circadian period length for rhythmic flies are indicated. Chi-square analysis indicated a highly significant (p<10^−8^) association between genotype and the percentages of rhythmic, weakly rhythmic, and arrhythmic adults. (C) Bar diagram of the average (±SEM) relative rhythmic power observed among the rhythmic plus weakly rhythmic flies for each genotype. The number of flies included in this analysis (n) is indicated for each condition. Because all but one *cyc^01^ (elav-Pdf)>-* flies were arrhythmic, a Mann-Whitney rank-sum test was performed to compare the effect on relative rhythmic power of the other two genotypes. As indicated, relative rhythmic power was significantly reduced in *cyc^01^ (elav-Pdf)>cyc* flies compared to *cyc^01^ elav>cyc* flies (***; p<10^−3^).
(PDF)
