# Supplementary figures and images for: Adult Circadian Behavior in Drosophila Requires Developmental Expression of cycle, But Not period
Source: PLoS Genet. 2011 Jul 7;7(7):e1002167. doi: 10.1371/journal.pgen.1002167 (PMC3131292; doi:10.1371/journal.pgen.1002167)

# S1A

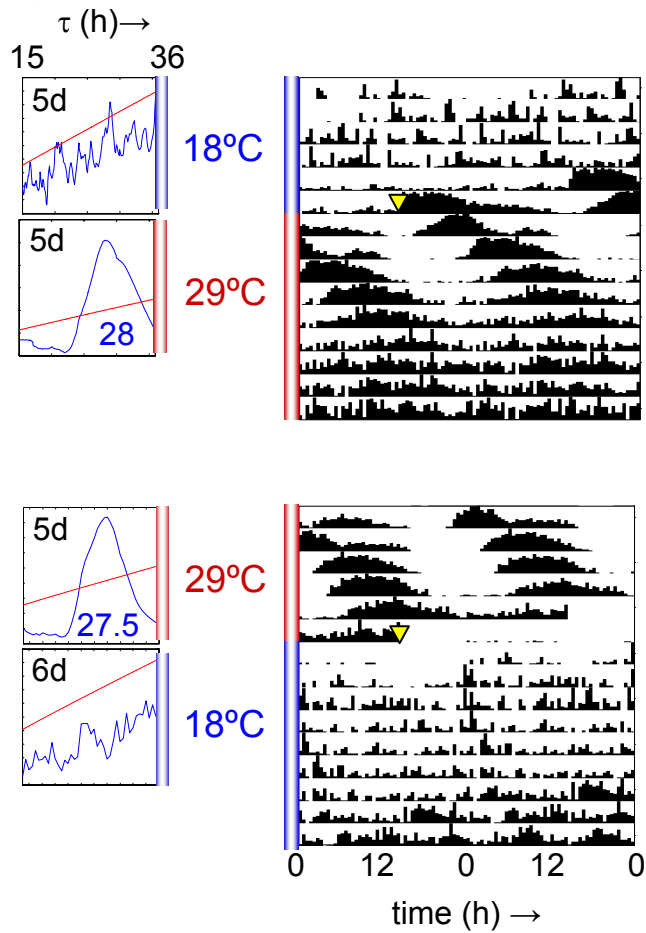

# B

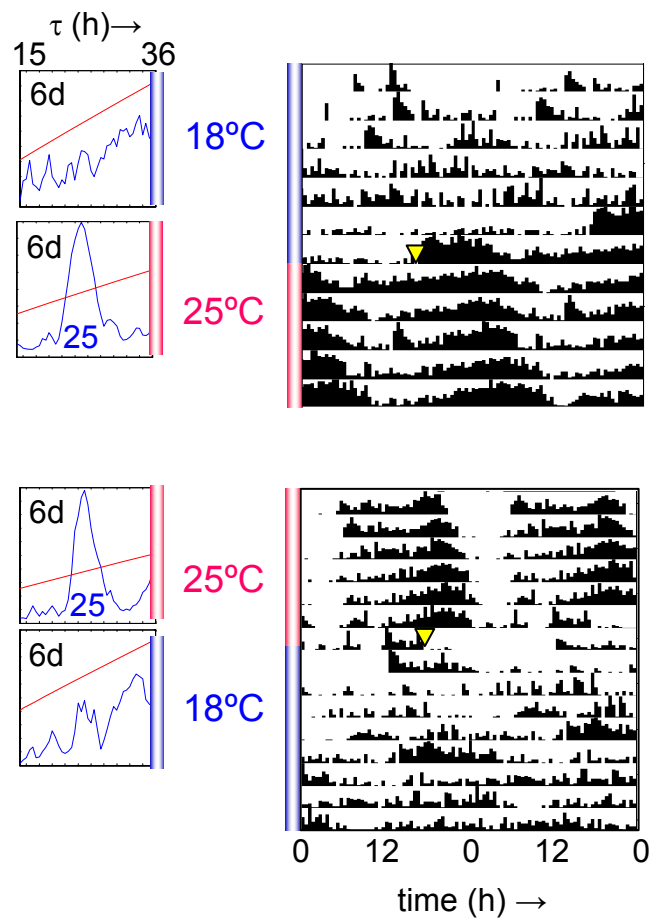

Supplement: Figure S1 — Conditional transgenic rescue of per01 arrhythmic behavior in male flies. (A,B) Median actogram and periodogram analyses for representative groups of male per01 [timP>per]ts flies (n = 8 in each case) that were obtained in parallel with the data for female flies in Figure 1B and 1C. Locomotor rhythms in DD are strongly reduced at the restrictive temperature (18°C) compared to permissive conditions (25°C, 29°C) and the lengthening period and weakening of rhythms in the long term observed at 29°C are likely attributable to the effects of excessive per expression. (PDF) [file pgen.1002167.s001.pdf]

Figure S4A

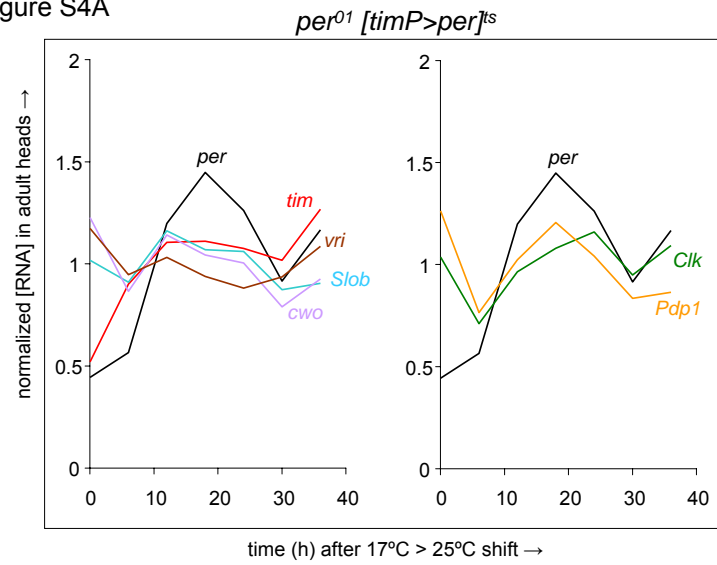

B

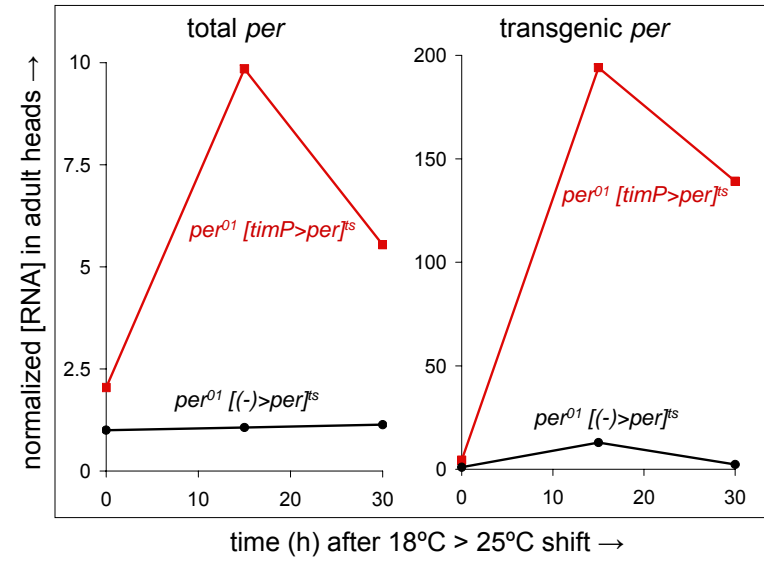

C

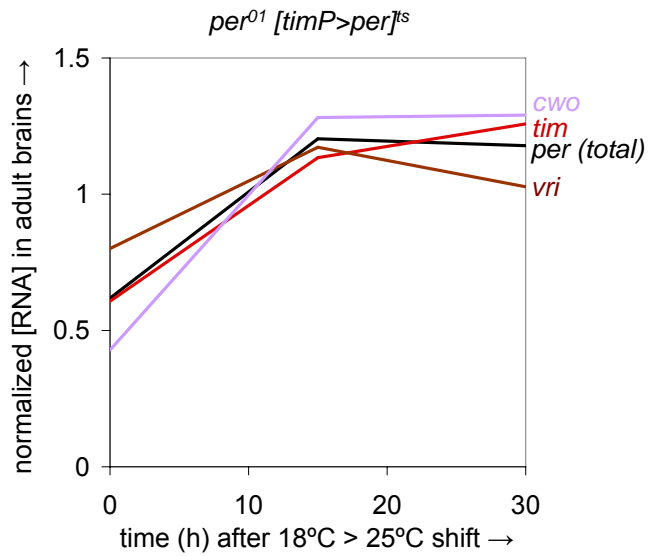

Supplement: Figure S4 — Conditional induction of clock gene mRNA expression in adult per01 [timP>per]ts heads and brains. (A) Total RNA extracted from adult fly heads collected immediately prior to or at 6,12,18,24,30, or 36 h after a shift from restrictive 17°C to permissive 25°C conditions in DD was analyzed on Northern blots for the expression of the indicated clock-controlled transcripts. The data for each transcript represent expression ratios relative to an internal control (rp49) that were normalized to the experimental average. The signal for per encompassed both the endogenous per01 and transgenic per transcripts and exhibited a temperature-dependent induction. Clock-controlled transcript profiles exhibited shallow amplitudes but maintained the expected relative phase relationships, with peak expression of tim, vri, cwo, and Slob peaking ahead of Pdp1, which in turn was phase-advanced relative to Clk. Similar observations were made for several independent experiments. (B) Quantitative Reverse Transcriptase PCR (qRT-PCR) analysis was performed using primers that were (right panel) or were not (left panel) selective for transgenic per relative to native per01 transcripts. Total RNA was extracted from the heads of per01 [timP>per]ts flies as well as controls lacking the tim(UAS)-Gal4 driver transgene (per01 [->per]ts) immediately before and at 15 and 30 h after a 18°C DD (restrictive) to 25°C DD (permissive) shift. Signals were quantified using the cycle threshold method [50] relative to rp49 transcript. Total per01/per transcript and transgenic per transcript were induced at 15 h in per01 [timP>per]ts heads approximately five-fold and more than forty-fold, respectively, while per transcript levels in per01 [->per]ts flies remained constitutively low. (C) Total RNA was extracted from dissected adult brains of per01 [timP>per]ts flies immediately prior to as well as at 15 and 30 h after a 18°C DD to 25°C DD shift. Signals in adult brains for clock genes (cwo, tim, vri) and total per01 [file pgen.1002167.s004.pdf]

Figure S6A

[*timP*>*per*]<sup>ts</sup>: 7x 17°C DL → **7x 17°C DD**

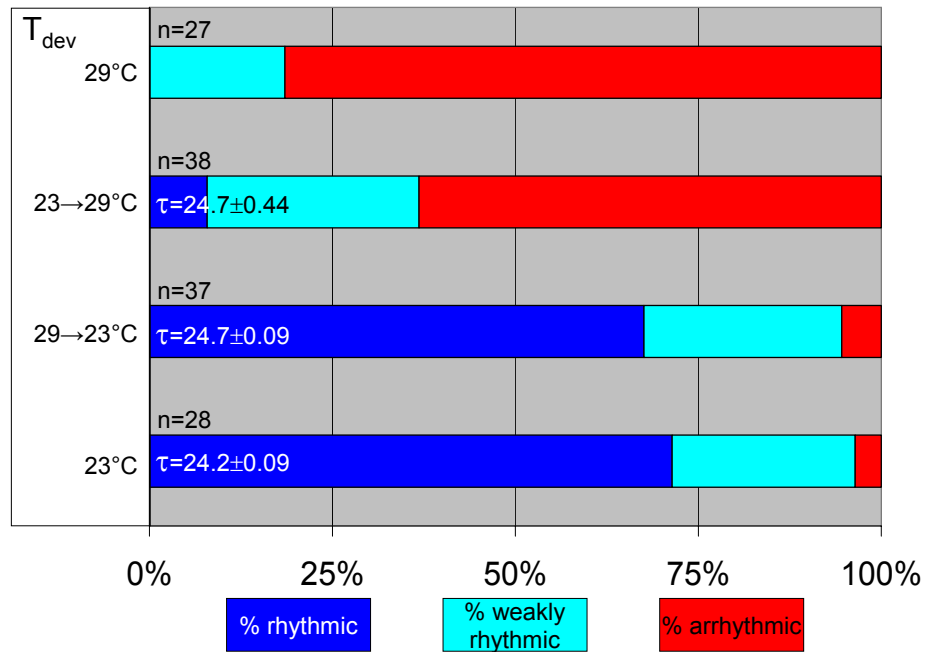

B

[*timP*>*per*]<sup>ts</sup>: 7x 17°C DL → **7x 17°C DD**

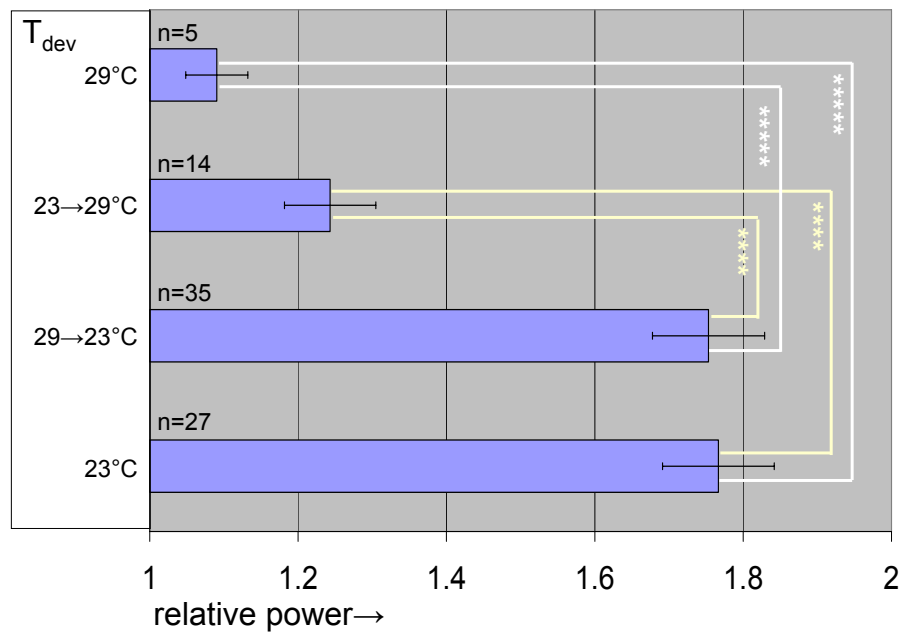

Supplement: Figure S6 — Over-expression of per in clock-bearing cells during metamorphosis disrupts locomotor activity rhythms in adult males. Circadian behavior of adult [timP>per]ts males under permissive conditions (17°C DD) was determined by the temperature of pupal and pharate adult development. While males raised at ambient temperature (∼23°C) or transferred from 29°C to ambient temperature as wandering larvae or prepupae were strongly rhythmic as adults at the permissive temperature, males exposed to restrictive (29°C DD) conditions throughout development or during the pupal and pharate adult stages exhibited arrhythmic adult locomotor behavior. (A) Stacked bar diagram representing the percentages of males with rhythmic, weakly rhythmic, or arrhythmic adult behavior at permissive conditions (17°C DD). Prior to measurement of adult locomotor activity during 17°C LD and subsequent 17°C DD (analyzed here) flies were raised at the indicated temperatures (Tdev): 23°C, 23→29°C (transferred as wandering larvae or prepupae from 23°C to 29°C), 29→23°C (transferred as wandering larvae or prepupae 29°C to 23°C), and 29°C. The numbers (n) of flies included for each condition are indicated as well as the average (±SEM) circadian period length for rhythmic flies. Chi-square analysis indicated a highly significant (p<10−13) association between developmental temperature and the percentages of rhythmic, weakly rhythmic, and arrhythmic adults. (B) Bar diagram of the average (±SEM) relative rhythmic power observed among the rhythmic plus weakly rhythmic male flies for each developmental condition. The number of flies included in this analysis (n) is indicated for each condition. The Welch test statistic indicated a highly significant association (p<10−8) of relative rhythmic power with developmental condition. Significant differences found by post-hoc Games-Howell tests for pairwise comparisons of developmental treatments indicated by (****) and (*****) represent p values smaller than 10−4 and 10−5. ( [file pgen.1002167.s006.pdf]

Figure S7A

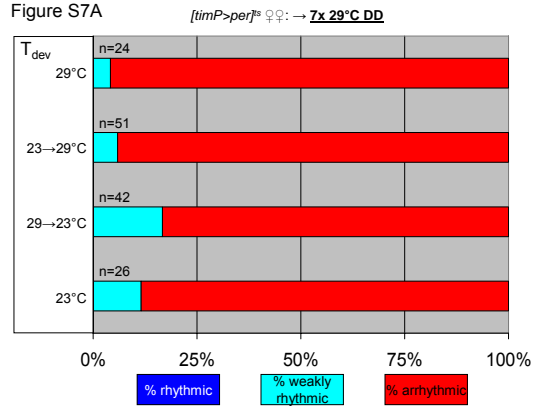

B

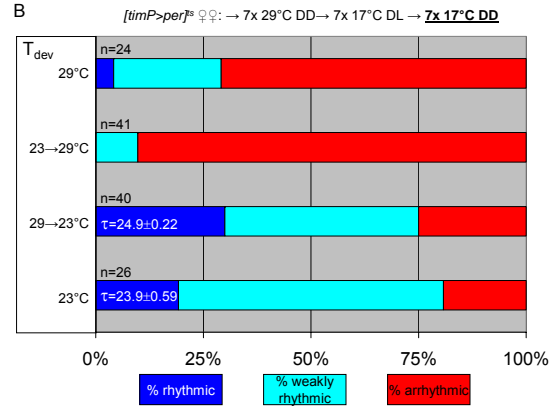

C

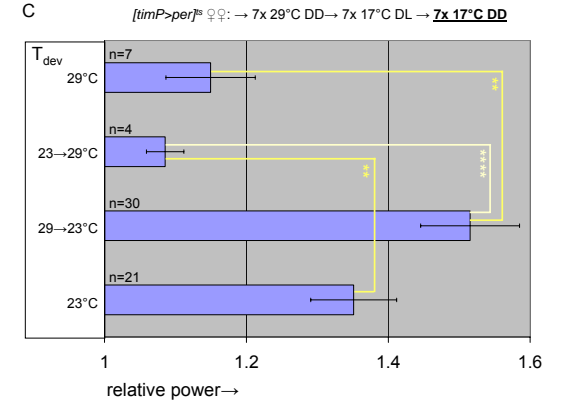

D

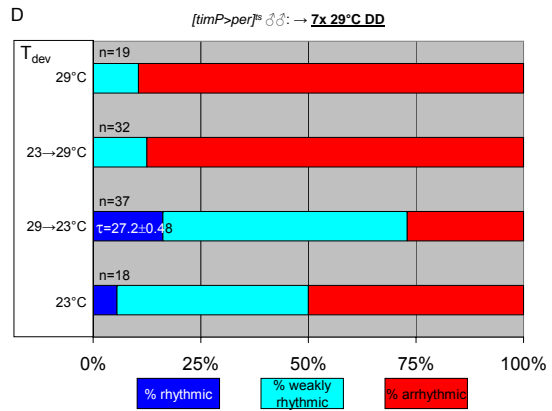

E

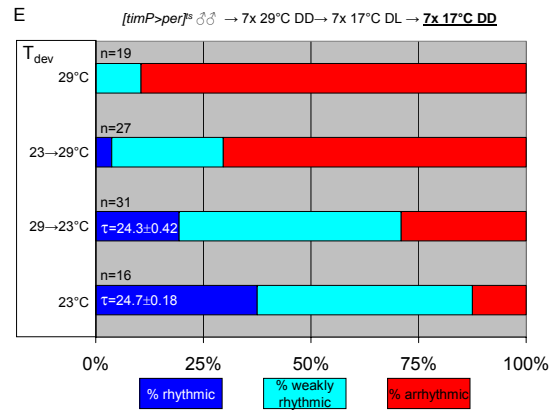

F

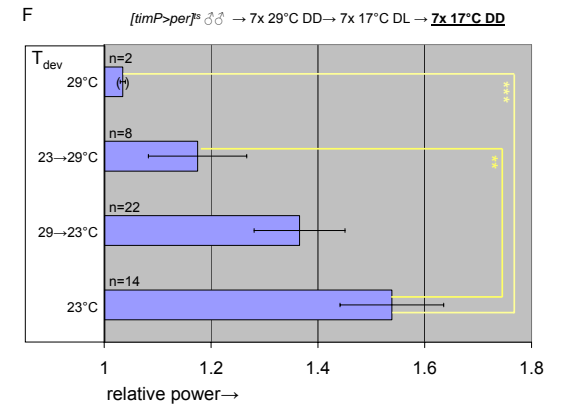

Supplement: Figure S7 — Developmental over-expression of per affects rhythmicity and power of adult circadian behavior. Circadian locomotor activity was analyzed for [timP>per]ts adult females (A–C) and males (D–F) that were raised either under restrictive conditions (29°C) or permissive conditions (∼23°C) or transferred from restrictive to permissive conditions, or vice versa, at the wandering larva/prepupa stage. (A,B,D,E) Stacked bar diagrams representing the percentages of rhythmic, weakly rhythmic, or arrhythmic flies at restrictive conditions (29°C DD) (A,D) or subsequent permissive conditions (17°C DD after 7 d at 17°C LD) (B,E). Prior to analysis of adult locomotor activity flies were raised at the indicated temperatures (Tdev): 23°C, 23→29°C (transferred as wandering larvae/prepupae from 23°C to 29°C), 29→23°C (transferred as wandering larvae/prepupae 29°C to 23°C), and 29°C. Average(±SEM) circadian period length is indicated for rhythmic flies. (A) Females were essentially arrhythmic at 29°C regardless of developmental treatment (Chi-square p = 0.25). (D) In contrast, males, which have a relatively higher dosage of Gal80ts (see Materials and Methods), showed a highly significant association between developmental temperature and rhythmicity at 29°C (Chi-square p<10−5) and exhibited long-period rhythms at 29°C following development under permissive conditions. (B,E) Both genders demonstrated a highly significant correlation between developmental treatment and rhythmicity at 17°C during the last step of the experiment (Chi-square females p<10−9, males p<10−5) when flies exposed to permissive conditions during metamorphosis showed circadian period lengths approaching 24-h. (C,F) Bar diagrams of the average(±SEM) relative rhythmic power observed among the rhythmic plus weakly rhythmic flies at 17°C DD for each developmental condition. For 29°C-raised males (n = 2) the range rather than the SEM is indicated in (F). The Welch test statistic indicated significant associations between rel [file pgen.1002167.s007.pdf]

Figure S8

$[timP>per]^{ts}$

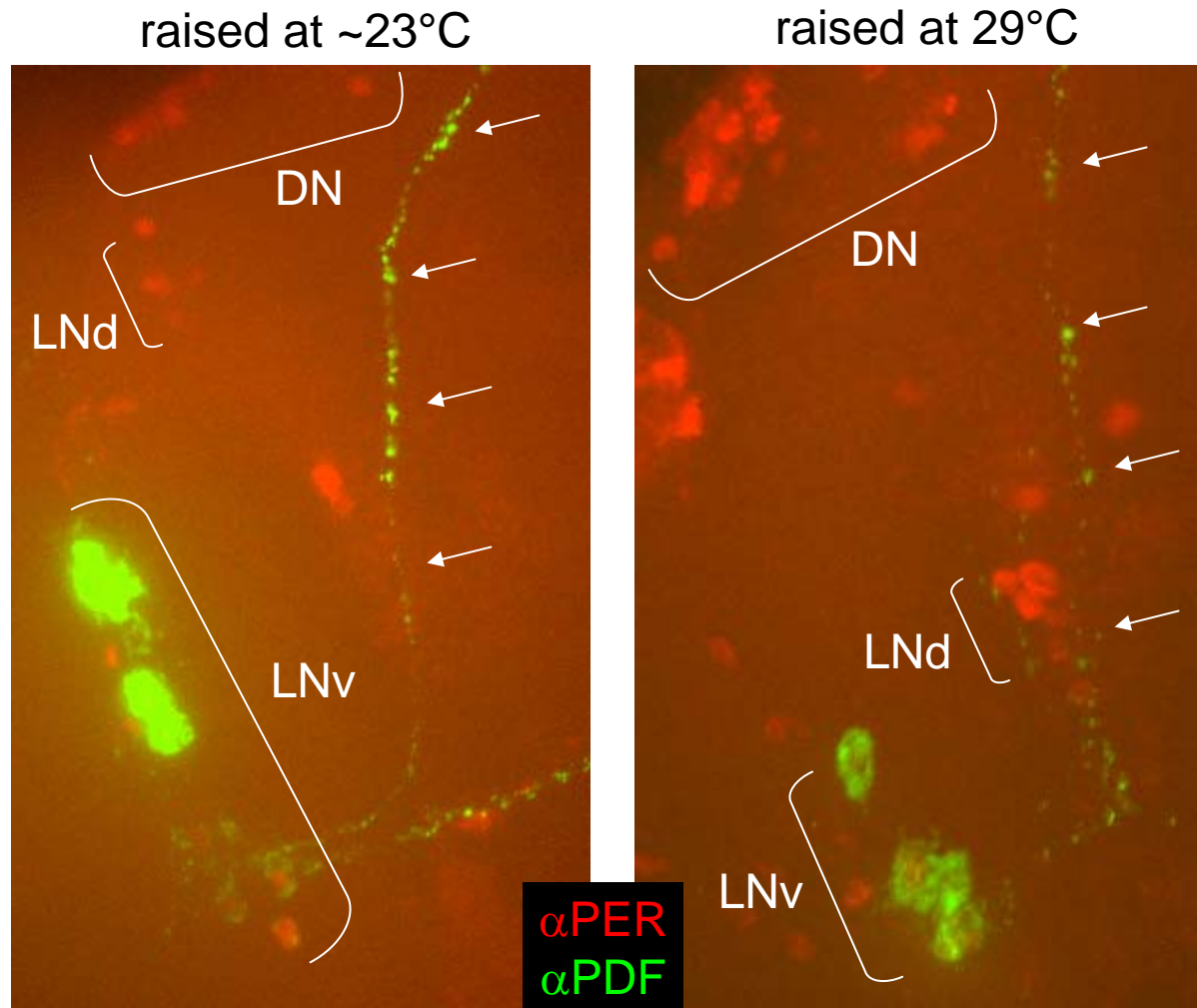

Supplement: Figure S8 — DN and LNd subsets of clock neurons as well as PDF-positive dorsal projections persist after developmental per over-expression. DN and LNd clock neurons were detected by anti-PER immunofluorescence in adult brains of both ∼23°C and 29°C-raised [timP>per]ts flies (experiment described in Figure 5). Representative images are shown. PER signal is weaker in the brain of the ∼23°C-raised fly because it was taken from the ZT4 time point, while the image for the 29°C-raised fly is from ZT22. LNv clock neurons are identified based on co-staining with anti-PDF antibody. Note the presence of PDF-stained dorsal projections from the LNvs in both brains (indicated by the white arrows). (PDF) [file pgen.1002167.s008.pdf]

Figure S10A

*cyc<sup>01</sup> [elav>cyc]<sup>ts</sup>: 7x 25°C DL → **7x 29°C DD***

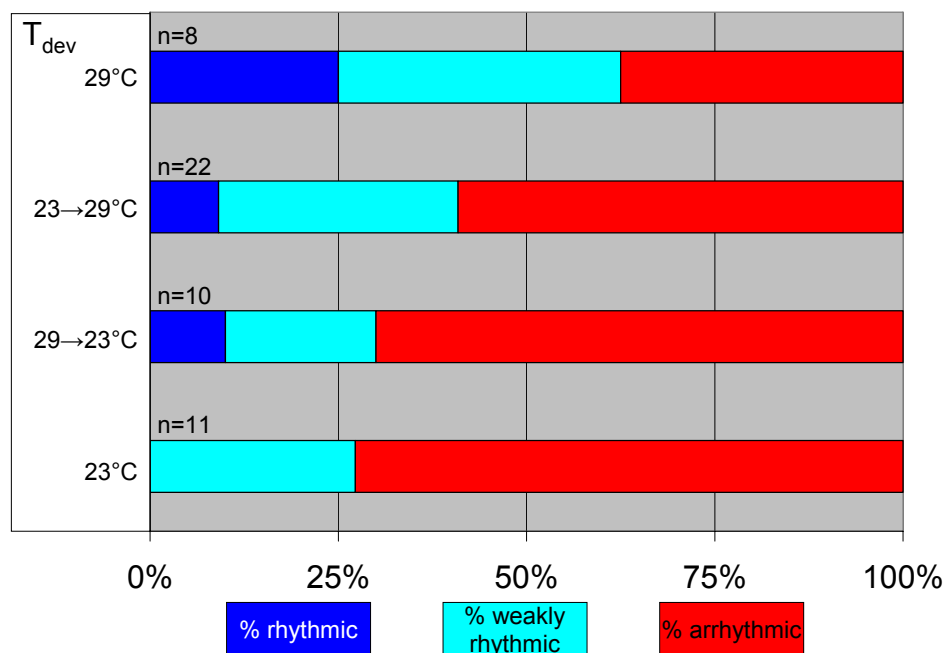

B

*cyc<sup>01</sup> [elav>cyc]<sup>ts</sup>: 7x 25°C DL → **7x 29°C DD***

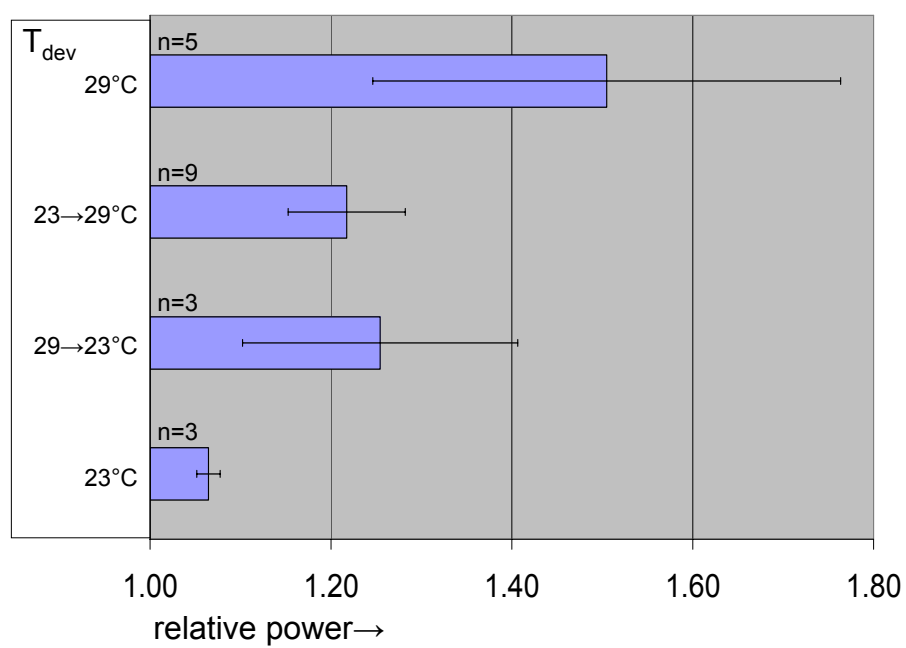

Supplement: Figure S10 — Association between developmental cyc expression and transgenic rescue of behavioral arrhythmia in adult males. (A) Stacked bar diagram representing the percentages of male flies with rhythmic, weakly rhythmic, or arrhythmic adult behavior at permissive conditions (29°C DD) following prior exposure to 7 LD days at 25°C. Flies were raised at the indicated temperatures (Tdev): 23°C, 23→29°C (transferred as wandering larvae or prepupae from 23°C to 29°C), 29→23°C (transferred as wandering larvae or prepupae 29°C to 23°C), and 29°C prior to analysis of adult locomotor activity during 25°C LD and subsequent 29°C DD conditions. The numbers (n) of flies included for each condition are indicated as well as the average (±SEM) circadian period length for rhythmic flies. Although the trends in rhythmicity relative to developmental conditions resemble the associations found in females chi-square analysis did not demonstrate a significant (p = 0.11) association in males. (B) Bar diagram of the average (±SEM) relative rhythmic power observed among the rhythmic plus weakly rhythmic flies for each developmental condition. The number of flies included in this analysis (n) is indicated for each condition. Again, trends of relative rhythmic power relative to developmental condition resemble the associations found in female flies, but the Welch test statistic did not demonstrate a significant association of relative rhythmic power with developmental condition (p = 0.15). (PDF) [file pgen.1002167.s010.pdf]

Figure S11A

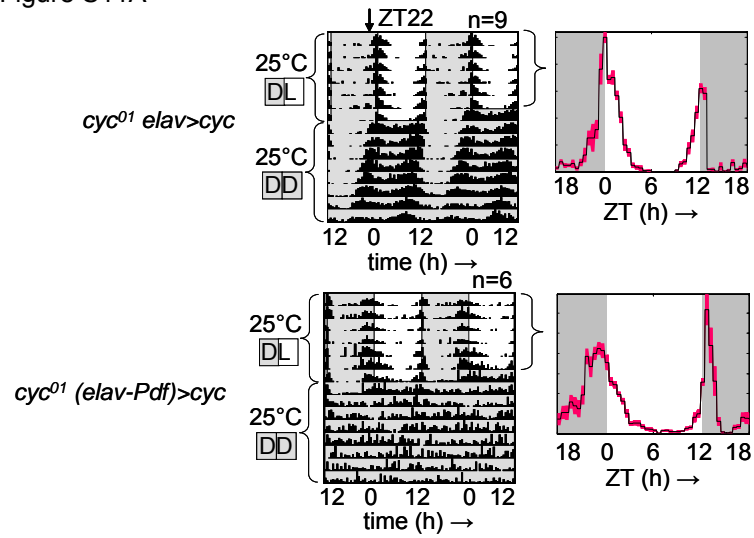

B

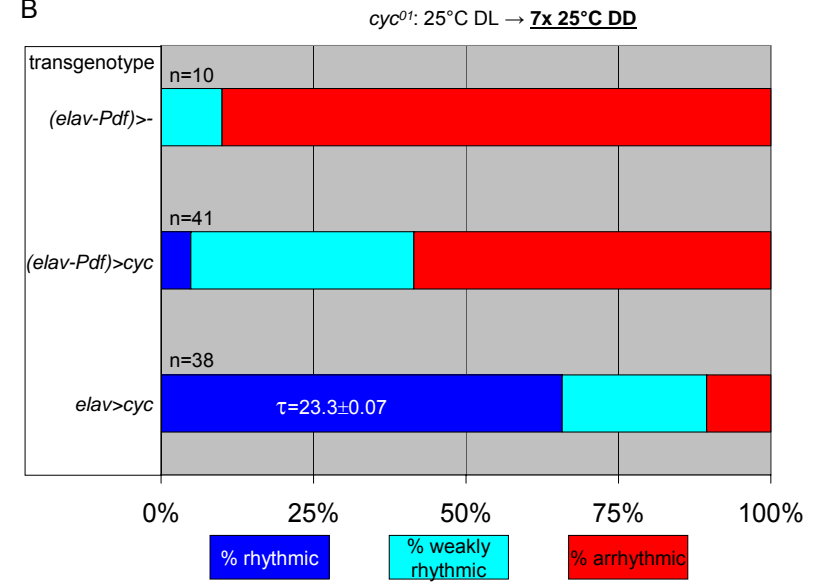

C

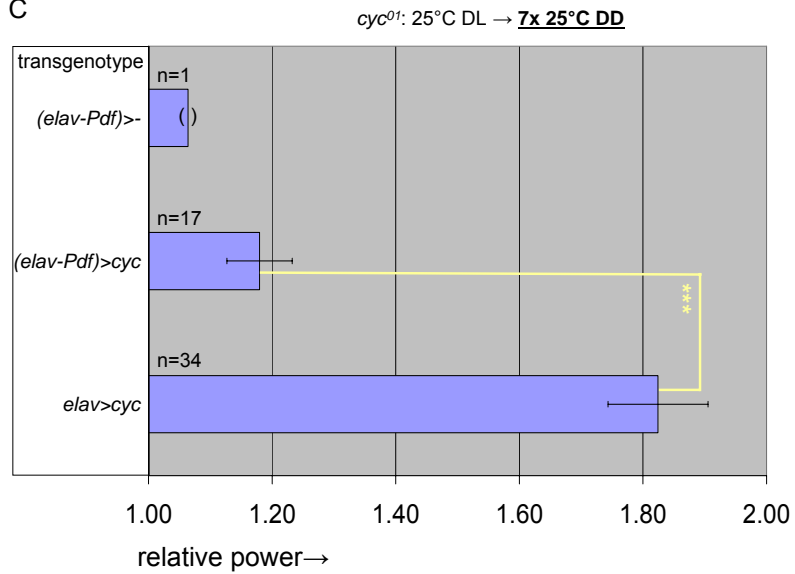

Supplement: Figure S11 — Male cyc01 (elav-Pdf)>cyc flies show behavioral arrhythmia. (A) Example actograms (left) and LD activity profiles (right) representing median locomotor behavior for male cyc01 elav>cyc versus cyc01 (elav-Pdf)>cyc flies. Note that cyc01 (elav-Pdf)>cyc flies exhibit loss of free running rhythms in DD as well as increased activity in anticipation of lights-on and loss of activity in anticipation of lights-off in LD. (B) Stacked bar diagram representing the percentages of cyc01 elav>cyc, cyc01 (elav-Pdf)>cyc, and cyc01 (elav-Pdf)>- male flies with rhythmic, weakly rhythmic, or arrhythmic adult behavior at 25°C DD. The number of flies (n) as well as the average (±SEM) circadian period length for rhythmic flies are indicated. Chi-square analysis indicated a highly significant (p<10−8) association between genotype and the percentages of rhythmic, weakly rhythmic, and arrhythmic adults. (C) Bar diagram of the average (±SEM) relative rhythmic power observed among the rhythmic plus weakly rhythmic flies for each genotype. The number of flies included in this analysis (n) is indicated for each condition. Because all but one cyc01 (elav-Pdf)>- flies were arrhythmic, a Mann-Whitney rank-sum test was performed to compare the effect on relative rhythmic power of the other two genotypes. As indicated, relative rhythmic power was significantly reduced in cyc01 (elav-Pdf)>cyc flies compared to cyc01 elav>cyc flies (***; p<10−3). (PDF) [file pgen.1002167.s011.pdf]
