# Supplementary material for: Adult Circadian Behavior in Drosophila Requires Developmental Expression of cycle, But Not period
Source: PLoS Genet. 2011 Jul 7;7(7):e1002167. doi: 10.1371/journal.pgen.1002167 (PMC3131292; doi:10.1371/journal.pgen.1002167)
Supplement: Figure S2 — Quantitative analysis of adult circadian behavior in per01 [timP>per]ts flies across a range of temperatures. Both male and female adult per01 [timP>per]ts flies exhibited temperature-dependent locomotor activity rhythms. The stacked bar diagrams (A,C) represent the percentages of female (A) and male (C) per01 [timP>per]ts with rhythmic, weakly rhythmic, or arrhythmic adult locomotor behavior at different experimental temperatures. Rhythmicity was determined for individual flies by chi-square periodogram analysis of 5-d intervals at the indicated temperatures in constant darkness. The numbers (n) of flies included for each condition are indicated as well as the average (±SEM) circadian period length for rhythmic flies. Chi-square analyses indicated highly significant associations (females p<10−15; males p<10−18) between experimental temperature and the percentages of rhythmic, weakly rhythmic, and arrhythmic adults. The bar diagrams (B,D) correspond to the average (±SEM) relative rhythmic power observed among the rhythmic plus weakly rhythmic female (B) and male (D) flies for each experimental temperature. The number of flies included in this analysis (n) is indicated for each condition. Welch test analyses indicated highly significant associations (females p<10−9; males p<10−10) of relative rhythmic power with experimental condition. Significant differences found by post-hoc Games-Howell tests for pairwise comparisons of developmental treatments indicated by (*), (**), (***), (****), and (*****) represent p values smaller than 0.05,10−2,10−3,10−4, and 10−5, respectively. (PDF) [file pgen.1002167.s002.pdf]

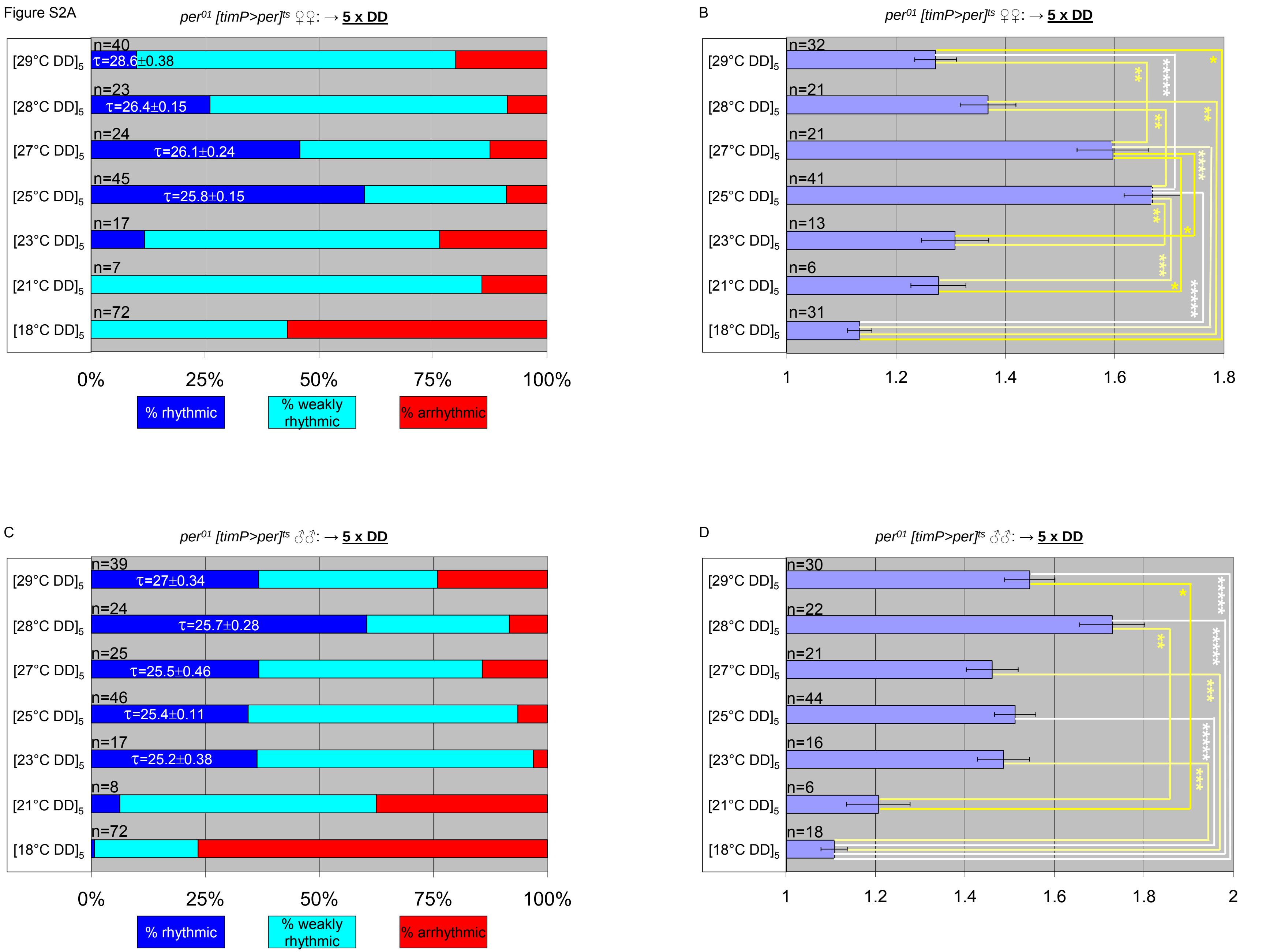

Figure S2A
per01 [timP>per]ts ♀♀: → 5 x DD
[29°C DD]5
[28°C DD]5
[27°C DD]5
[25°C DD]5
[23°C DD]5
[21°C DD]5
[18°C DD]5
n=40
τ=28.6±0.38
n=23
τ=26.4±0.15
n=24
τ=26.1±0.24
n=45
τ=25.8±0.15
n=17
n=7
n=72
0%
25%
50%
75%
100%
% rhythmic
% weakly rhythmic
% arrhythmic
B
per01 [timP>per]ts ♀♀: → 5 x DD
n=32
n=21
n=21
n=41
n=13
n=6
n=31
1
1.2
1.4
1.6
1.8
C
per01 [timP>per]ts ♂♂: → 5 x DD
n=39
τ=27±0.34
n=24
τ=25.7±0.28
n=25
τ=25.5±0.46
n=46
τ=25.4±0.11
n=17
τ=25.2±0.38
n=8
n=72
D
per01 [timP>per]ts ♂♂: → 5 x DD
n=30
n=22
n=21
n=44
n=16
n=6
n=18
1
1.2
1.4
1.6
1.8
2
