# Supplementary material for: Adult Circadian Behavior in Drosophila Requires Developmental Expression of cycle, But Not period
Source: PLoS Genet. 2011 Jul 7;7(7):e1002167. doi: 10.1371/journal.pgen.1002167 (PMC3131292; doi:10.1371/journal.pgen.1002167)
Supplement: Figure S3 — Comparison of temperature-dependent locomotor behavior of flies with conditional rescue of per01 to that of wild-type flies. (A–C) Analyses of locomotor behavior of per01 [timP>per]ts (conditional per01 rescue) and Canton-S (wild-type) flies in 5-d intervals in constant darkness at 18°C (A), 25°C (B), and 29°C (C). Top row: Actograms for representative individual flies showing locomotor activity across Circadian Time (CT) in constant darkness. Middle row: Chi-square periodogram data for the 5-d intervals and flies represented in the top row. Significant circadian period lengths (τ; p<0.01) are indicated (blue type face) as well as classification into rhythmic (R), weakly rhythmic (WR) and arrhythmic (AR) flies. Bottom row: Average circadian activity profiles were generated from the average data of 8 (or 7) flies over the 5-d intervals. The profiles are plotted relative to the circadian period lengths detected in the average data. If no significant periodicity was found, (A: column 1,2) the profiles were plotted over 24 h. (D) The stacked bar diagram represents a comparison of the distribution of rhythmic, weakly rhythmic, or arrhythmic per01 [timP>per]ts flies at 18°C, 25°C, and 29°C relative to wild-type (Canton-S) controls. The numbers (n) of flies included for each condition are indicated as well as the average (±SEM) circadian period length for rhythmic flies. Chi square analyses revealed significant differences in rhythmicity at 18°C and 29°C, but not 25°C, with p-values smaller than 10−4 and 10−5 indicated by (****) and (*****), respectively. In addition, Mann-Whitney rank-sum tests comparing circadian period length at 25°C and 29°C between rhythmic per01 [timP>per]ts and wild-type control flies indicated significantly longer periods (p<0.001) for the former. (PDF) [file pgen.1002167.s003.pdf]

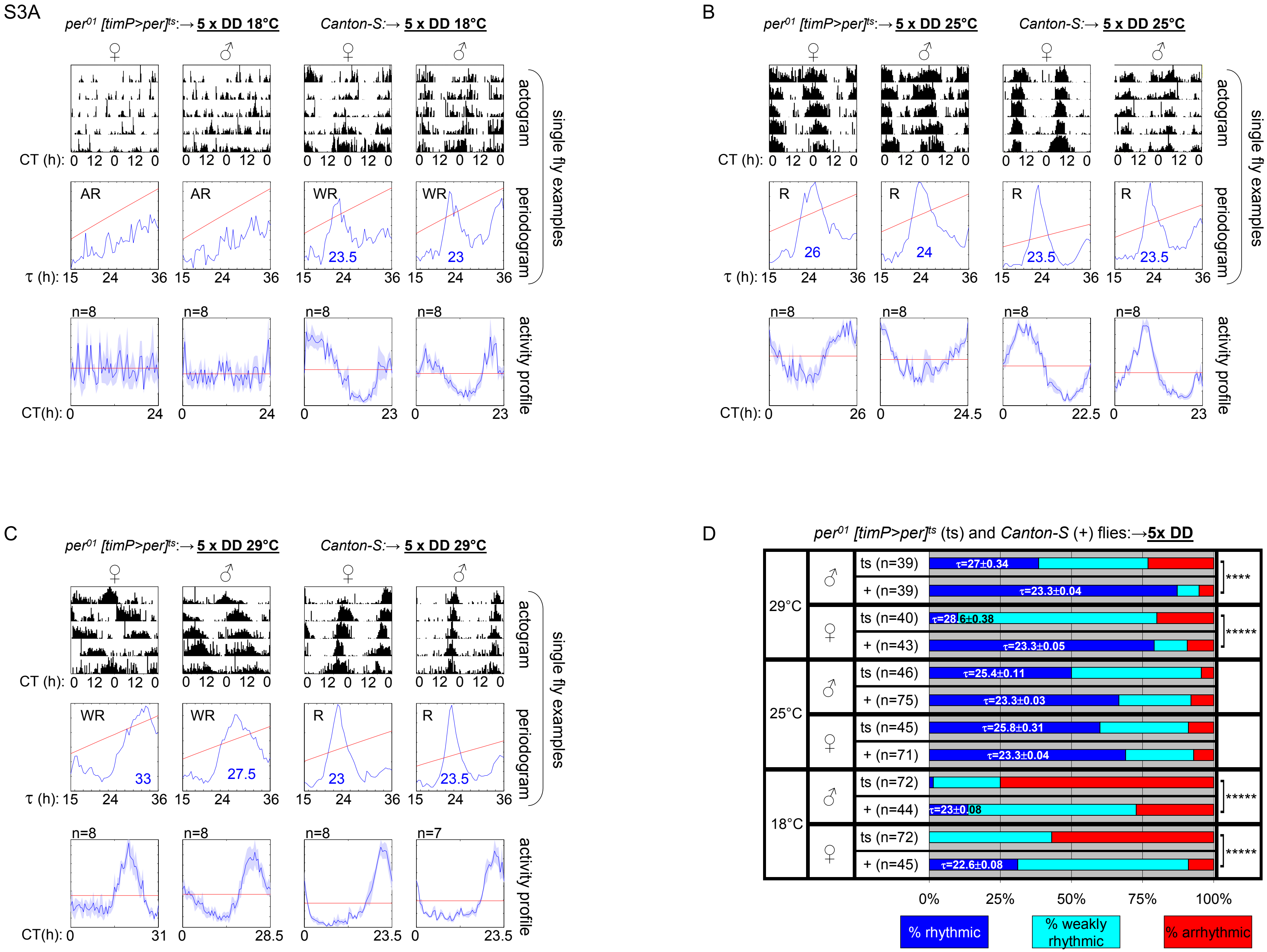
S3A
per01 [timP>per]ts:→ 5 x DD 18°C
Canton-S:→ 5 x DD 18°C
B
per01 [timP>per]ts:→ 5 x DD 25°C
Canton-S:→ 5 x DD 25°C
C
per01 [timP>per]ts:→ 5 x DD 29°C
Canton-S:→ 5 x DD 29°C
♀
♂
CT (h): 0 12 0 12 0
τ (h): 15 24 36
CT(h): 0
actogram
periodogram
activity profile
single fly examples
AR
WR
R
n=8
n=7
D
per01 [timP>per]ts (ts) and Canton-S (+) flies:→5x DD
29°C
25°C
18°C
ts (n=39)
+ (n=39)
ts (n=40)
+ (n=43)
ts (n=46)
+ (n=75)
ts (n=45)
+ (n=71)
ts (n=72)
+ (n=44)
ts (n=72)
+ (n=45)
τ=27±0.34
τ=23.3±0.04
τ=28.6±0.38
τ=23.3±0.05
τ=25.4±0.11
τ=23.3±0.03
τ=25.8±0.31
τ=23.3±0.04
τ=23±0.08
τ=22.6±0.08
****
*****
0% 25% 50% 75% 100%
% rhythmic
% weakly rhythmic
% arrhythmic
