# Supplementary material for: Adult Circadian Behavior in Drosophila Requires Developmental Expression of cycle, But Not period
Source: PLoS Genet. 2011 Jul 7;7(7):e1002167. doi: 10.1371/journal.pgen.1002167 (PMC3131292; doi:10.1371/journal.pgen.1002167)
Supplement: Figure S5 — Rhythmicity and power of circadian locomotor activity are conditionally rescued in per01 [timP>per]ts adults raised at restrictive conditions. Adult locomotor behavior was examined in female (A,B) and male (C,D) adult per01 [timP>per]ts flies raised under restrictive conditions (17°C LL) at restrictive (17°C DD) and subsequent permissive conditions (25°C DD). (A,C) Stacked bar diagrams representing the percentages of flies with rhythmic, weakly rhythmic, or arrhythmic adult behavior during 7 d at 17°C DD versus 7 d at 25°C DD. The numbers (n) of flies included for each condition are indicated as well as the average (±SEM) circadian period length for rhythmic flies. Chi-square analyses indicated a highly significant association between experimental condition and the percentages of rhythmic, weakly rhythmic, and arrhythmic females (p<10−6) and males (p<10−4). (B,D) Bar diagrams of the average (±SEM) relative rhythmic power observed among the rhythmic plus weakly rhythmic flies for each developmental condition. The number of flies included in this analysis (n) is indicated for each condition. Statistical analyses (Mann-Whitney rank-sum test) indicated a significant association between relative rhythmic power and experimental condition in both females (**; p<10−2) and males (***; p<10−3). (PDF) [file pgen.1002167.s005.pdf]

Figure S5A

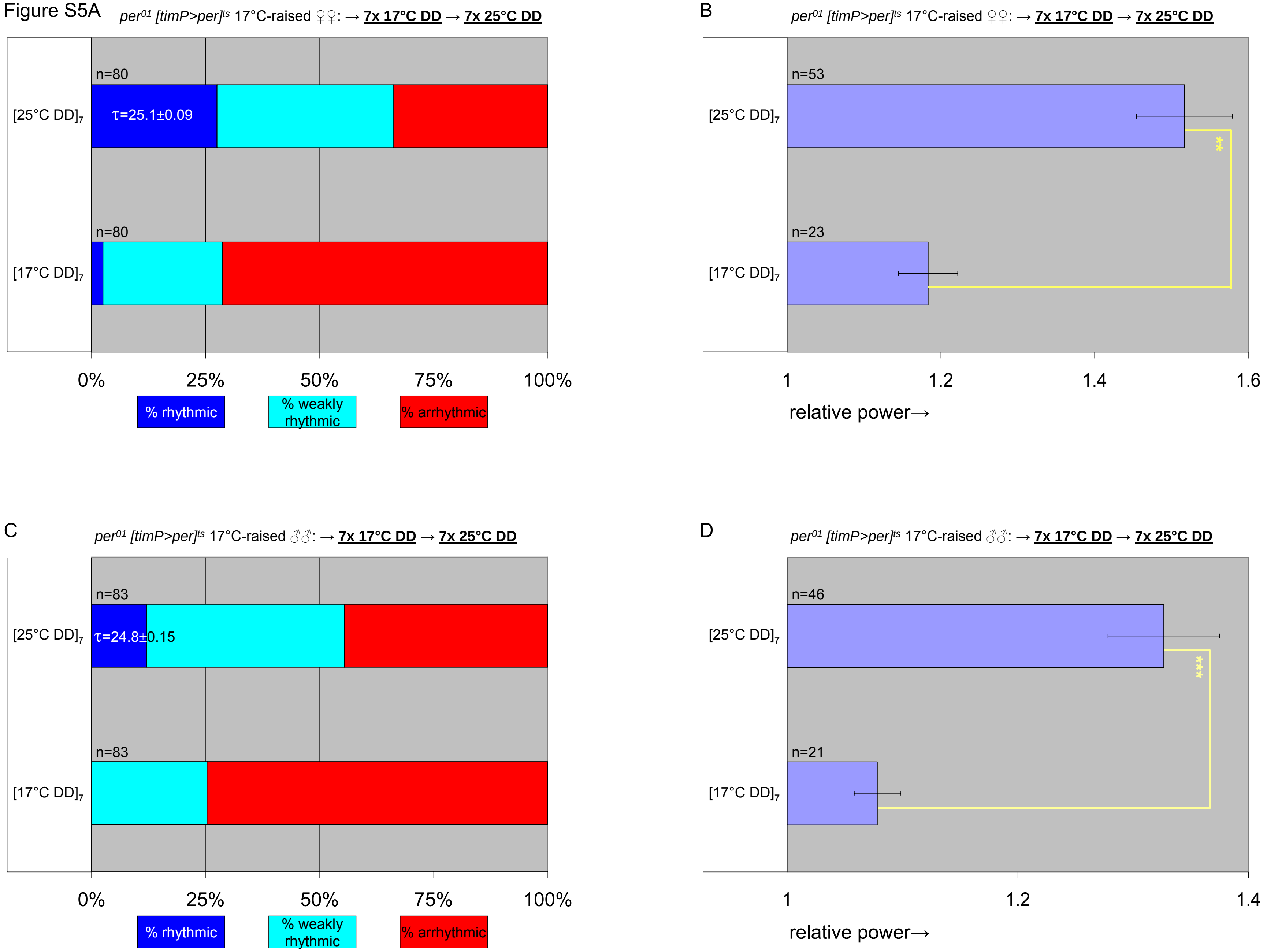

per01 [timP>per]ts 17°C-raised ♀♀: → 7x 17°C DD → 7x 25°C DD
n=80
[25°C DD]7
τ=25.1±0.09
n=80
[17°C DD]7
0%
25%
50%
75%
100%
% rhythmic
% weakly rhythmic
% arrhythmic
B
per01 [timP>per]ts 17°C-raised ♀♀: → 7x 17°C DD → 7x 25°C DD
n=53
[25°C DD]7
n=23
[17°C DD]7
1
1.2
1.4
1.6
relative power→
C
per01 [timP>per]ts 17°C-raised ♂♂: → 7x 17°C DD → 7x 25°C DD
n=83
[25°C DD]7
τ=24.8±0.15
n=83
[17°C DD]7
0%
25%
50%
75%
100%
% rhythmic
% weakly rhythmic
% arrhythmic
D
per01 [timP>per]ts 17°C-raised ♂♂: → 7x 17°C DD → 7x 25°C DD
n=46
[25°C DD]7
n=21
[17°C DD]7
1
1.2
1.4
relative power→
