# Supplementary material for: Adult Circadian Behavior in Drosophila Requires Developmental Expression of cycle, But Not period
Source: PLoS Genet. 2011 Jul 7;7(7):e1002167. doi: 10.1371/journal.pgen.1002167 (PMC3131292; doi:10.1371/journal.pgen.1002167)
Supplement: Figure S9 — Clock function is conditional in adult cyc01 [elav>cyc]ts males raised at the permissive condition. Quantitative analysis of adult circadian behavior in 29°C-raised cyc01 [elav>cyc]ts male flies at either permissive (29°C) versus two restrictive conditions (17°C, 18°C) (A,B) or permissive (29°C) versus restrictive (17°C) conditions following adult exposure to restrictive conditions (≥3 days 17C) (C,D). The stacked bar diagrams (A,C) represent the percentages of 29°C-raised male cyc01 [elav>cyc]ts flies with rhythmic, weakly rhythmic, or arrhythmic adult locomotor behavior. Rhythmicity was determined for individual flies by chi-square periodogram analysis of 7 d intervals at the indicated temperatures in constant darkness. The numbers (n) of flies included for each condition are indicated as well as the average (±SEM) circadian period length for rhythmic flies. Chi-square analyses indicated significant associations between experimental temperature and the percentages of rhythmic, weakly rhythmic, and arrhythmic adults (p<10−3 (A); p<10−13 (C)). The bar diagrams (B,D) correspond to the average (±SEM) relative rhythmic power observed among the rhythmic plus weakly rhythmic flies for each experimental condition. The number of flies included in this analysis (n) is indicated for each condition. Because there were only two observations for rhythmic/weakly rhythmic males at 18°C the range rather than the average±SEM is indicated. (B) Welch test analyses indicated a significant association of relative rhythmic power with experimental condition (p<10−2). A significant difference was found by post-hoc Games-Howell test for pairwise comparison of males at 29°C versus 18°C (**; p<0.02). (D) Statistical analysis (Mann-Whitney rank-sum) indicated a significant association between relative rhythmic power and experimental condition (***; p<10−3). (PDF) [file pgen.1002167.s009.pdf]

Figure S9A

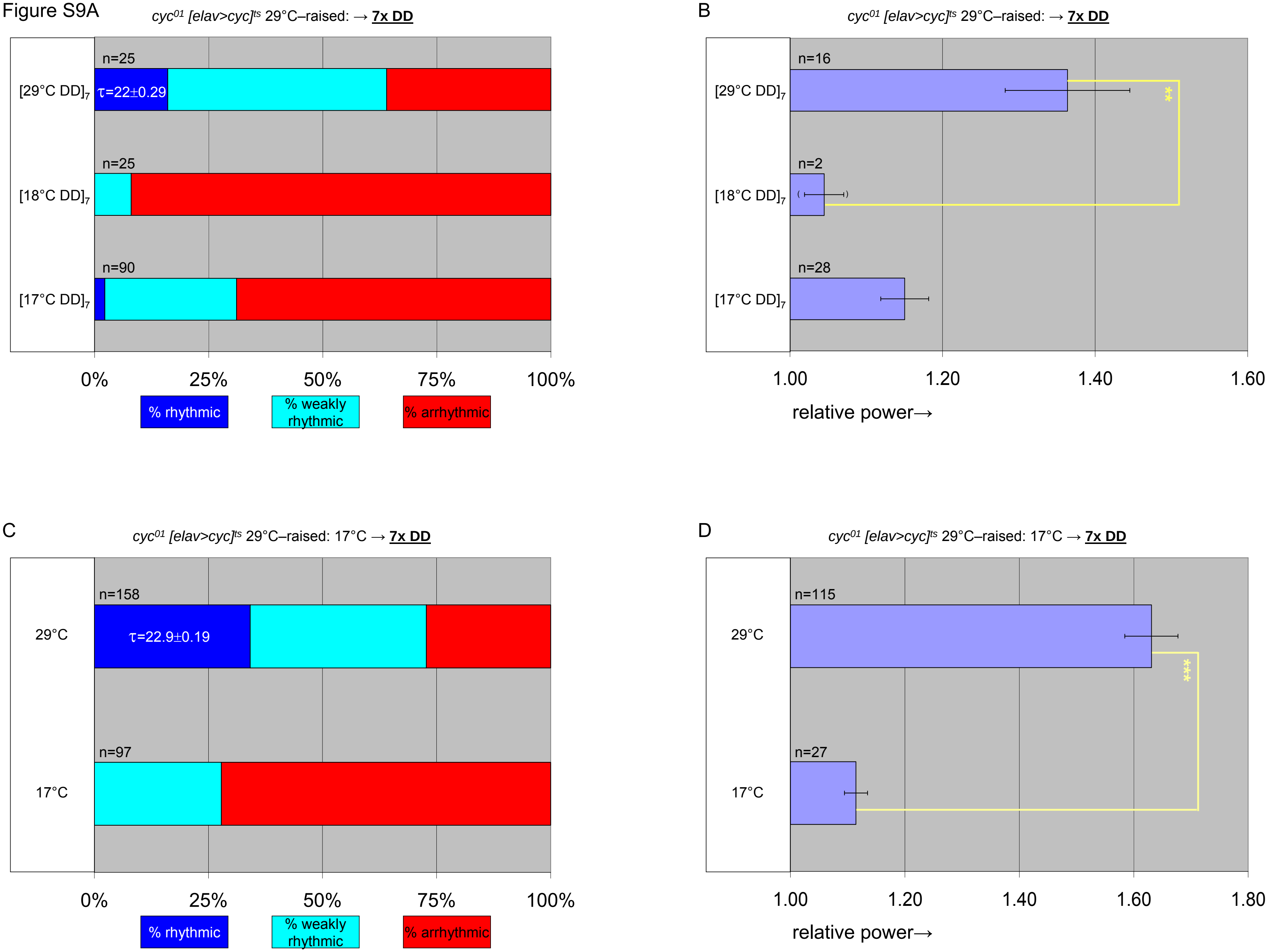

cyc01 [elav>cyc]ts 29°C–raised: → 7x DD
n=25
[29°C DD]7
τ=22±0.29
n=25
[18°C DD]7
n=90
[17°C DD]7
0%
25%
50%
75%
100%
% rhythmic
% weakly rhythmic
% arrhythmic
B
cyc01 [elav>cyc]ts 29°C–raised: → 7x DD
n=16
[29°C DD]7
**
n=2
[18°C DD]7
n=28
[17°C DD]7
1.00
1.20
1.40
1.60
relative power→
C
cyc01 [elav>cyc]ts 29°C–raised: 17°C → 7x DD
n=158
29°C
τ=22.9±0.19
n=97
17°C
0%
25%
50%
75%
100%
% rhythmic
% weakly rhythmic
% arrhythmic
D
cyc01 [elav>cyc]ts 29°C–raised: 17°C → 7x DD
n=115
29°C
***
n=27
17°C
1.00
1.20
1.40
1.60
1.80
relative power→
